# Tribochemistry, Mechanical Alloying, Mechanochemistry: What is in a Name?

**DOI:** 10.3389/fchem.2021.685789

**Published:** 2021-05-26

**Authors:** Adam A. L. Michalchuk, Elena V. Boldyreva, Ana M. Belenguer, Franziska Emmerling, Vladimir V. Boldyrev

**Affiliations:** ^1^ Federal Institute for Materials Research and Testing (BAM), Berlin, Germany; ^2^ Novosibirsk State University, Novosibirsk, Russia; ^3^ Boreskov Institute of Catalysis SB RAS, Novosibirsk, Russia; ^4^ Yusef Hamied Department of Chemistry, University of Cambridge, Cambridge, United Kingdom; ^5^ Voevodski Institute of Chemical Kinetics and Combustion SB RAS, Novosibirsk, Russia

**Keywords:** mechanochemistry, tribochemistry, mechanical alloying, tribology, mechanical activation, nomenclature, mechanochemical pictographs

## Abstract

Over the decades, the application of mechanical force to influence chemical reactions has been called by various names: mechanochemistry, tribochemistry, mechanical alloying, to name but a few. The evolution of these terms has largely mirrored the understanding of the field. But what is meant by these terms, why have they evolved, and does it really matter how a process is called? Which parameters should be defined to describe unambiguously the experimental conditions such that others can reproduce the results, or to allow a meaningful comparison between processes explored under different conditions? Can the information on the process be encoded in a clear, concise, and self-explanatory way? We address these questions in this Opinion contribution, which we hope will spark timely and constructive discussion across the international mechanochemical community.

## Introduction

Chemical transformations initiated by mechanical energy appear to be the first reactions that humans learned to induce and control, even before thermal reactions were possible. In fact, the first combustion reactions were produced through mechanical action: by percussion or by friction, *i.e*. they were *mechanochemical* or *tribochemical*, if modern terminology were used. Throughout human history, mechanically induced chemical reactions have accompanied many significant technological advances. For example, since the discovery of black powder in *ca.* 220 B.C.E., explosives have allowed the advent of mining and have facilitated the construction of cities and infrastructures. More recently, the continued development of mechanochemistry promises to revolutionize the chemical industry, providing synthetic routes devoid of environmentally harmful solvents (James et al., 2012; Baláž et al., 2013). The potential for mechanochemistry to have paradigm-changing impact across the chemical sciences has placed the field amongst IUPAC’s ‘10 chemical innovations that will change our world’ (Gomollón-Bel, 2019).

The earliest written record of a mechanochemical transformation seems to be that by Theophrastus of Eresus, in his book “On Stones” of *ca.* 315 B.C.E (Takacs, 2000). Theophrastus describes the reduction of cinnabar to mercury through grinding using a copper mortar and pestle. Although grinding and milling were used extensively over the centuries for the processing of grains, minerals, and even pharmaceuticals, mention of mechanochemical processes in scientific literature did not reappear until the 19th century. These early reports include those by Faraday (1820) on the dehydration of crystal hydrates (Heinicke, 1984; Takacs, 2013), Carey-Lee (1866) on the decomposition of silver, gold, and mercury halides on grinding (Carey-Lea, 1892; Carey-Lea, 1894), and by both Ling and Baker (1893) (Heinicke, 1984; Takacs, 2013) and Flavitsky (Flavitsky, 1902; Flavitsky, 1909) who described organic chemical reactions upon grinding. The attention of mechanochemical investigation soon expanded to a wide range of material types, and explored an array of phenomena including the initiation of explosives by impact and friction (Bowden et al., 1947; Bowden and Gurton, 1949; Fox, 1975; Aduev et al., 1999), and the mechanical decomposition of polymers (Butyagin, 1971; Polukhina and Baramboim, 1975; Oprea, 1979; Sohma, 1989; Delogu et al., 2017). Similarly, mechanochemical investigation into areas including the chemical processes accompanying mining, metallurgy, and the manufacturing of various oxide and chalcogenide materials became a prominent direction of research (Senna, 1993; Butyagin, 1994; Boldyrev, 1996; Fernández-Bertran, 1999; Steinike and Tkáčová, 2000; Senna, 2001; Epelak, 2002; Boldyrev, 2006; Buyanov et al., 2009; Šepelák et al., 2012; Šepelák et al., 2013), expanding toward the preparation and processing of fine chemicals and pharmaceuticals (Baba et al., 1990; Kuzuya et al., 1991; Otsuka et al., 1994; Dubinskaya, 1999; Kondo, 2000; Boldyrev, 2004; Otsuka et al., 2011). The 20^th^ century represents a period of remarkable development of the fundamental aspects of mechanochemistry and of significant progress in scaling mechanochemical reactions toward real-world industrial applications. Although progress in mechanochemistry through the 20th century was dominated by studies of metals, inorganic compounds, materials, and catalysts, significant advances were also made in the mechanochemistry of organic polymers and drug compounds and formulations (Boldyrev and Avvakumov, 1967; Butyagin, 1994; Boldyrev, 1996; Fernández-Bertran, 1999; Steinike and Tkáčová, 2000; Watanabe et al., 2001; Watanabe et al., 2002; Watanabe et al., 2003; Boldyrev, 2004; Boldyrev, 2006; Buyanov et al., 2009). A number of dedicated texts on the historical development of mechanochemistry are available elsewhere (Boldyrev and Tkáčová, 2000; Takacs, 2013; Boldyrev, 2018).

To date, mechanochemical approaches being applied to transformations from across the chemical sciences have been reported, spanning from the synthesis of inorganic and organic compounds [including those as complex as peptides (Hernández et al., 2017; Maurin et al., 2017)] through to the preparation of large porous frameworks such as metal-organic frameworks [MOFs(Stolar et al., 2017; Stolar and Užarević, 2020; Wang et al., 2020; Zhou et al., 2020; Głowniak et al., 2021; Stolar et al., 2021)]. Moreover, the scale of mechanochemical reactions has ranged from the mechanical manipulation of single atoms and molecules (predominantly, synthetic and natural polymers) using atomic force microscopy (Kaupp, 2009; Ribas-Arino and Marx, 2012; Makarov, 2016; Li et al., 2017) to the induction of reactions in multi-component inorganic and organic powder mixtures in ball milling reactors or extruders (Iwasaki et al., 2010; Am Ende et al., 2014; Oliveira et al., 2017; Andersen and Mack, 2018b; Egleston et al., 2020). Alongside synthetic covalent chemical reactions, a wide range of supramolecular assemblies have been also prepared by mechanical treatment, including cocrystals and salts (Myz et al., 2009; Weyna et al., 2009; Friščić, 2012; Myz et al., 2012; Braga et al., 2013; Hasa et al., 2015), as well as non-covalently bound mechano-composites such as drug delivery devices comprising active pharmaceutical ingredients with excipients (Shakhtshneider et al., 2007; Shakhtshneider et al., 2014a; Shakhtshneider et al., 2014b; Lomovsky et al., 2017; Ogienko et al., 2018; Bychkov et al., 2019; Adekenov et al., 2020; Skripkina et al., 2020). Moreover, many organic mechanochemical syntheses have been successfully scaled-up (Iwasaki et al., 2010; Am Ende et al., 2014; Crawford et al., 2017; Trofimova et al., 2018; Stolar et al., 2019; Baláž et al., 2020; Crawford et al., 2020; He et al., 2020; Titi et al., 2020; Stolar et al., 2021), offering a direct route to translate mechanochemical synthesis toward industrial scale applications. It can be therefore expected that many industrial syntheses will be soon conducted mechanochemically, alongside the long-standing technologies of inorganic mechanosynthesis and of powder processing *e.g.* in the pharmaceutical, pulp-paper, mining, explosives, and food processing industries.

**FIGURE 1:**
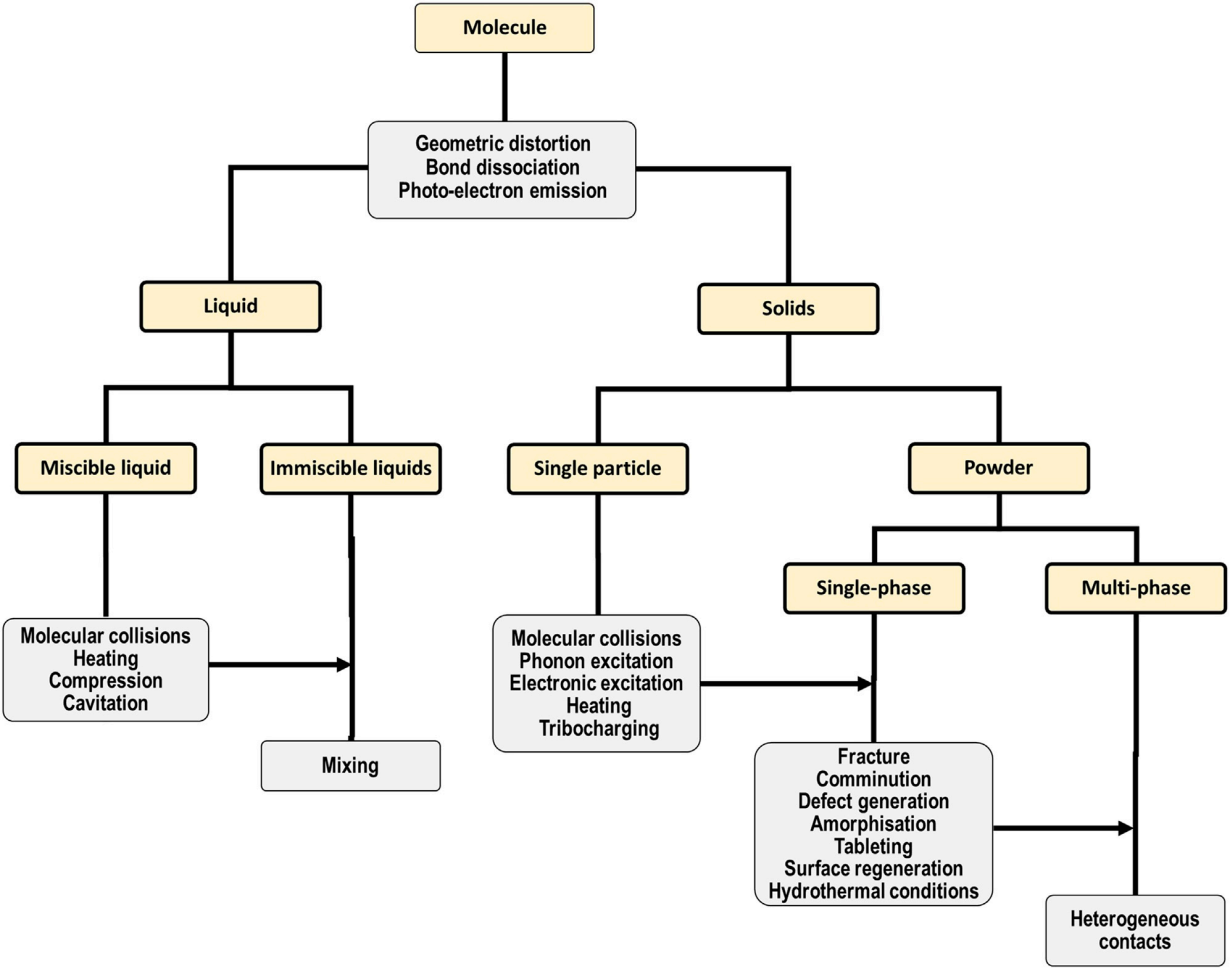
Hierarchical representation of the major effects (gray) of mechanical action on different systems, ranging from single molecules to multi-phase solid powder mixtures (gold).

Indeed, this enormous range of applications for mechanochemical preparations demands that the processes which govern their transformation must be equally diverse, as shown in the hierarchical diagram in Figure 1. There is some elegance to this complexity: many of the processes which govern mechanochemical reactions of complex systems can be largely deconstructed into some combination of the elementary processes which occur in simpler systems. For example, mechanical treatment of a single powder particle will still involve geometric distortion of its molecular substituents (Haruta et al., 2019), and there remains the potential for molecular or atomic electronic excitation/emission processes to occur. This behavior is clear for example in high pressure experiments of molecular solids, wherein mechanical action of the bulk lattice yields geometric (Fabbiani et al., 2005) and electronic distortions (Poręba et al., 2019) or excitations (Tulip and Bates, 2009) at the molecular or atomic level(Boldyreva, 2019; Katrusiak, 2019; Zakharov and Boldyreva, 2019). The dynamical stressing (compression or shearing) of solids can also cause chemical species within the solid state to approach each other at high velocities, akin to molecular collisions in fluids. Such dynamical interactions can occur either within a single particle (Landerville et al., 2009; Zhang et al., 2015; Steele et al., 2020), or at inter-particle contacts (Ferguson et al., 2019). These solid state “molecular collisions” have been suggested as the origin of slip-induced “hot spots” (Zhang et al., 2008; Zhang et al., 2015; Zhang et al., 2018), or even covalent bond formation (Engelke and Blais, 1994).

That said, with increasing complexity of the system, many more and new pathways exist by which mechanical action can exert influence. This hierarchical phenomenology is again exemplified by the study of high-pressure phenomena in molecular solids. Mechanical force not only affects molecular geometry but can also influence the intermolecular non-covalent interactions, leading to changes in crystal packing (polymorphism). By manipulating crystal packing, mechanical force thus offers a route to modify bulk physical properties such as lattice stability, melting temperatures, and compressibility. Thus, although many of the elementary stages of a solution-based chemical reaction may still apply to mechanochemical reactions, many additional elementary stages must be also considered to fully account for the mechanism of mechanochemical transformations. Regardless of the exact elementary processes involved, mechanical action drives the system out of equilibrium, which may involve a classical phase transition (Drebushchak et al., 2011) or *via* some other transient metastable state (Butyagin, 1971). This intermediate state can endure *e.g.* under continuous mechanical action and provide modified tribological properties (Prentice et al., 2020; Reddyhoff et al., 2021), or relax when the mechanical perturbation is released to yield a reaction product (Boldyrev, 2006). Owing to the significant kinetic barriers in solid state reactions, this reaction product may not be always the most thermodynamically stable one (*i.e.* the global minimum of the system), but it will always be the most accessible product under the given mechanochemical conditions (Lin and Nadiv, 1979). Identifying, understanding, predicting, and ultimately controlling these pathways leading to the mechanical manipulation of matter is crucial, should mechanochemical approaches ever become equally controllable as the well-developed aspects of solution and gas-phase reactivity.

Deconvolution of these complex phenomena requires the use of a common language which allows the effective communication of the process being discussed. Only in this way can we hope for a concerted and coherent effort towards elucidating mechanochemical reaction mechanisms and driving forces, and therefore gain control over these reactions to make them possible to reproduce and scale. Despite decades of mechanochemical research, the need to agree on using certain terms, on how to define accurately and unambiguously the experimental procedures, and how to present the results have been not widely seen as necessary until very recently. For almost a century, the mechanochemical community remained relatively small, although it covered a diverse range of fields. Researchers knew not only the scientific research of the others but often knew each other personally. The basics of mechanochemistry, as well as the experimental and computational protocols were discussed regularly in original publications, and at many dedicated mechanochemical seminars and conferences. The developments in the field were regularly summarized in thoroughly detailed monographs and reviews, that have now become seminal (Thießen, 1965; Avvakumov, 1972; Boldyrev, 1972; Boldyrev, 1983; Heinicke, 1984; Tkáčová, 1989; Avvakumov et al., 2001; Suryanarayana et al., 2001; Delogu et al., 2004a; Boldyrev, 2006; Butyagin, 2006; Zyryanov, 2008; Buyanov et al., 2009). The foundation of the International Mechanochemical Association (IMA) (International Mechanochemical Association, 2020) under the guide of IUPAC in 1989, was an important event that marked the formation of a mature scientific community with a common language and a clear vision of the scientific field.

As an increasing number of groups have begun in recent years to enter the field of mechanochemistry independently of IMA, the quickly growing community has since become scientifically heterogeneous. In contrast to its original composition, the mechanochemical community is now becoming enriched with researchers from very different backgrounds and expertize, many of them being originally experts not in the solid-state, but in solution-based chemistry. This diversification in its membership has brought with it many new and exciting research challenges, leading to a much greater impact of mechanochemistry than ever before. Yet one cannot ignore the fact that with diversification of the community comes an expanding breadth of specialized scientific languages. As a consequence, the heterogenous community may not always understand each other effectively, or may become increasingly unaware of the mechanochemical knowledge that has been accumulated in early publications, that itself can be perceived as being written in “another scientific language”. There is the real danger that the lack of use of a common scientific language can prevent the community from meeting the challenge of constructing the “Tower of Mechanochemistry”, as it did millennia ago in relation to the Tower of Babel.

## What is in a Name?

Anthropologists argue that humankind evolved due to our capacity to conceive abstract phenomena and communicate these phenomena through complexity of language. In this light, it is no wonder that philosophers have attached such significance to the selection and connotation of words. In Plato’s famous dialogue Cratylus, he argues: “a name is an instrument of teaching and of distinguishing natures, as the shuttle is of distinguishing the threads of the web” (Plato, 1961). The chemical sciences have followed true to Plato’s logic. Chemical reactions are denoted according to the type of energy used to initiate a chemical reaction, their nature is revealed through their name: thermo-chemistry, electro-chemistry, magneto-chemistry, photo-chemistry, and radiation-chemistry. Thus, adequately naming a chemical reaction *requires an elementary understanding of the underlying chemical and physical processes*.

In the early 20^th^ century, Ostwald (Ostwald, 1919) noted reports that existing nomenclature in the chemical sciences did not fully represent the true nature of all observed chemical reactions, for Carey-Lea demonstrated a unique outcome of thermal- and mechanochemical reactions in metal halides (Carey-Lea, 1892; Carey-Lea, 1894). Correspondingly, Ostwald introduced in his 1919 textbook the term *mechanochemistry* to describe reactions *in any state of aggregation* which are initiated by mechanical force (impact and friction). A more specific term—*tribochemistry*—was subsequently proposed to denote only those chemical and physico-chemical changes which occur *in solids* in response to mechanical energy (Heinicke, 1970). The term *tribochemistry* was used widely throughout the 20^th^ century in relation to processes that occur on grinding, ball milling, comminution, friction, wear, rubbing, and lubrication of solids. With growing diversity of tribochemical reactions, daughter terms became commonplace to facilitate more accurately the communication of the scientific work. These words included tribocatalysis, triboelectrochemistry, tribosorption, tribodiffusion, tribocorrosion, tribotechnology, tribomechanics, tribogalvanics, and tribometallurgy, each introducing specific subfields of tribology, the science uniting tribochemistry and tribophysics (Thiessen et al., 1966; Thiessen, 1974; Heinicke, 1984; Holmberg and Erdemir, 2017). A primary aim for introducing these “tribo” terms in addition to Ostwald’s term “mechanochemistry”, was to separate solid-state mechanochemistry from mechanically induced processes that occur in single molecules or liquids. In this way, physical phenomena induced by mechanical action in solids or at their surfaces, including phenomena like mechanical mixing and comminution, were to be denoted with a “tribo” prefix. All other mechanically induced phenomena were instead to be defined by a prefix “mechano”.

**FIGURE 2:**
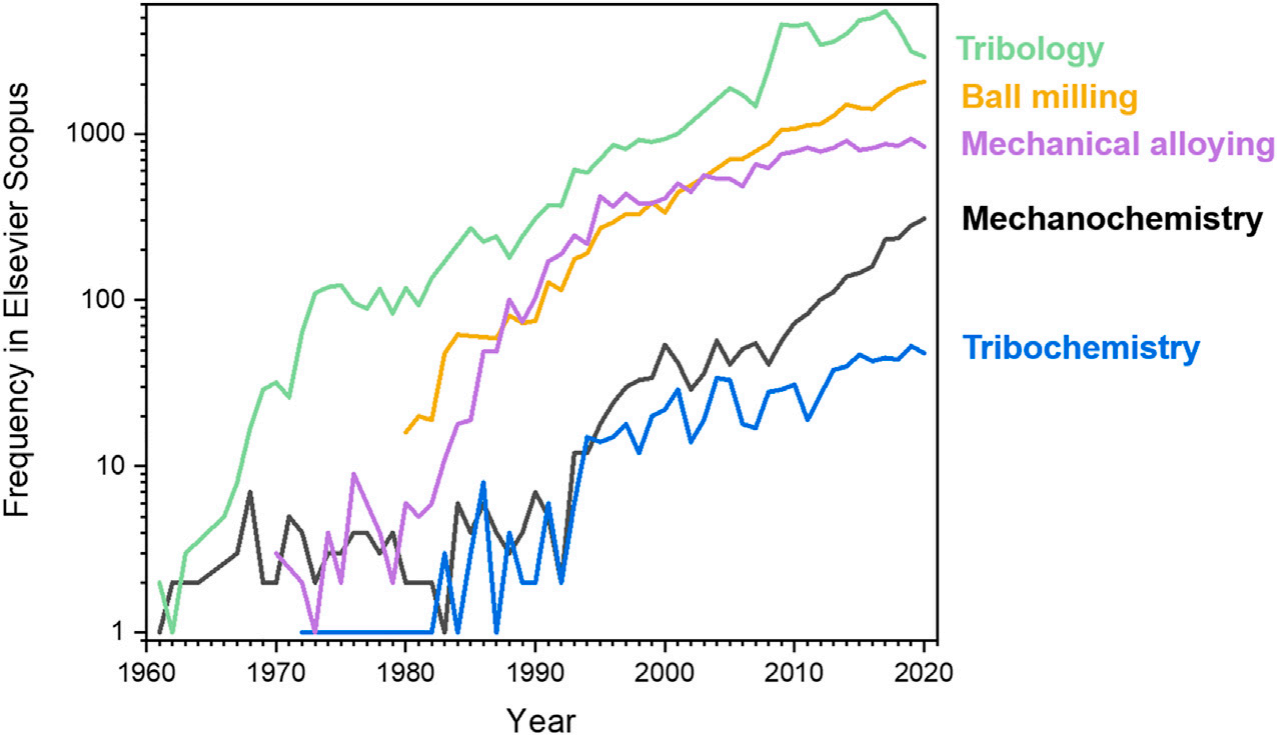
The number of papers in Scopus each year in which the terms related to chemical reactions of solids induced by mechanical treatment are used in the title, abstract or as keywords.

Despite growing popularity of tribological nomenclature, analogous terms prefixed by “mechano” remained in the literature as synonyms also for processes involving solids, including: mechanochemistry, mechanocatalysis, mechanocorrosion, and mechanotechnology. Moreover, the term *mechanical alloying* was introduced to define the process of forming intermetallic compounds and alloys by mechanical treatment of solid components, many of which could not be accessed by any other way than mechanical treatment (Suryanarayana et al., 2001; Benjamin and Volin, 1974; Koch, 1989; El-Eskandarany, 2020). Mechanical alloying processes are of great practical importance, which may account for the extensive publications of mechanical alloying studies and, consequently, of the very wide usage of this term in the scientific literature. The nomenclature regarding the mechanical manipulation of single molecules has remained much simpler. Only terms prefixed by “mechano” have been commonly used when discussing transformations of single-molecules induced by mechanical stretching of bonds, *e.g.* using an AFM cantilever (Kaupp, 2009; Ribas-Arino and Marx, 2012; Makarov, 2016; De Bo, 2018; Zhang et al., 2017; Beyer and Clausen-Schaumann, 2005), or when investigating biochemical and biophysical processes (Keller and Bustamante, 2000; Kushwaha and Peterman, 2020; Boocock et al., 2021). Figure 2 gives an idea of the relative frequencies of how the usage of different terms has changed with time. *Tribology*—the term introduced by Jost (Jost, 1990) in the 1960s—remains by far the most popular term to date, while *mechanochemistry* is much less used than *tribology*, or *mechanical alloying*. While the organic chemistry community appears to favor the term *mechanochemistry*, the terms *tribology* and *tribochemistry* are more popular amongst chemical engineers and the materials science community (Huq et al., 2020; Moshkovich et al., 2020; Moshkovich and Rapoport, 2020; Rosenkranz et al., 2020; Rosenkranz et al., 2021). In addition, the term *mechanical activation* is widely used in relation to thermal reactions that are facilitated by mechanical pre-treatment (Pavljukhin et al., 1983; Pavlyukhin et al., 1984; Boldyrev, 1996; Šepelák et al., 1996; Boldyrev, 1998; Boldyrev, 2006; Kumar et al., 2019; Singla et al., 2020).

*What is in a name? That which we call a rose by any other name would smell as sweet.* This famous phrase by W. Shakespeare serves an important lesson for science: the physical world does not depend on our description of it. Of course, our choice of nomenclature has no influence on the physical reality of chemical reactions. This nomenclature does, however, influence on our understanding, communication, and formulation of scientific principles. Nature is indifferent to our terminology. Scientists, in contrast, are prisoners to nomenclature. Without consistent and precise definition of scientific concepts, *“the rose*” may not smell as sweet.

Discussions of nomenclature in science has a long history. Most famous, perhaps, are the classifications of species proposed by Charles Darwin. The taxonomic classification of life demonstrates Plato’s reflection of a name in understanding the properties and connections between entities. Similar ontological classifications have been popular in chemistry throughout its history. For example, chemists routinely classify *interatomic interactions* according to an abstract definition of *bond order*: *single*, *double*, *triple*, etc. This precise nomenclature allows scientists to directly and unambiguously describe a characteristic of a *molecule* directly by the type of *bond*. Recently, a standard set of bond descriptions were suggested to define particular types of molecular interactions (Arunan et al., 2011; Desiraju et al., 2013; Aakeroy et al., 2019). Standardizing nomenclature has long been the focus of the IUPAC. More broadly, ensuring that well-defined and well-classified ontologies exist throughout the sciences is becoming increasingly recognized as the route to ensure Findable, Accessible, Interoperable, and Reusable (FAIR) scientific data (Hall and McMahon, 2016; Wilkinson et al., 2016).

So, what is in a name? Would that which we call “mechanochemistry” by any other name behave the same? Of course, the physical principles which govern mechanochemical reactions will behave independent of our chosen nomenclature, but will the conceptual constructions we use to rationalize, discuss, and direct scientific research be so resilient? The idea of complementing the term mechanochemistry by the term *tribology* (tribochemistry + tribophysics), to focus more on transformations involving solids (Jost, 1990), is clear and justified. In practice, the more general term *mechanochemistry* appears to survive. Moreover, it is increasingly used as a complete synonym of *tribology*, also when describing ball milling, grinding, and friction (Thiessen, 1986). This hazy nomenclature would not create much problem if it were always straightforward to identify which mechanically induced *physical* processes in solids were responsible for the *chemical* processes of bond cleavage and formation. This, however, is not the case. Defining accurately by its name the nature of a mechanically induced transformation has serious implications for the type and importance of physical processes which must be considered when seeking to understand mechanochemical transformations. Moreover, it is critically important to consider that molecules in the solid state cannot immediately react with one-another. Instead, some solid-state phenomenon must first occur which allows collisions not at the level of *particles* but at the level of *molecules and atoms.* The nature of this preliminary phenomenon depends on whether the reaction is mechano or tribochemical. For example, whereas mixing and comminution may be dominant preliminary phenomena in mechanochemical reactions, electrostatic charging or generation of defects certainly dominate many tribochemical reactions (Boldyrev, 2006). Hence, focus on chemical equilibria presented in terms of solution chemistry, where “one molecule transforms into another molecule”, are grossly oversimplified and neglect many of the critical physical phenomena which separates mechano/tribochemistry from solution chemistry.

Important phenomena such as triboelectric charging (Matsusaka et al., 2010; Cezan et al., 2019) and the mechanical generation of exposed surfaces (Belenguer et al., 2016; Schneider-Rauber et al., 2021) or defects are equally likely to occur in inorganic, organic, and polymeric compounds. It is probable that such phenomena involving defect formation (Boldyrev, 1973; Yelsukov et al., 2013) play a central role in most solid-state mechanochemical transformations, even if they are often overlooked and subsumed by explanations of “mere mixing”. Mechanically generated defects can range from radicals and the isomerization of molecules to extended stacking faults, dislocations, and the formation of shear-induced structures (*e.g.* shear bands). For example, it is known that grinding initially leads to particle size reduction (Lampronti et al., 2021), and ultimately (after a critical grinding or comminution limit is achieved) to the accumulation of defects within the solid structure (Boldyrev et al., 1996). This change in stress relaxation mechanism can lead to deep mechanical activation thereby greatly affecting the reactivity of the solid. Such effects have been suggested as being responsible for the extended induction periods observed in some mechanochemical reactions (Belenguer et al., 2019b). Mechanical treatment of a solid can also lead to the formation of mesophases, wherein superstructures within the phase become disordered to different degrees. Although this phenomenon appears to be more common in organic solids (Rybin et al., 2014; Descamps and Willart, 2016b; Shalaev et al., 2016; Rybin et al., 2019), it draws analogies to the mechanochemistry of inorganic compounds as well. For example, mechanical treatment can induce disordering of different sublattices such as the disordering or re-ordering of metal cations between octahedral and tetrahedral positions in spinels (Pavlyukhin et al., 1984; Tkáčová et al., 1996; Šepelák et al., 1996; Sepelak et al., 1997; Šepelák et al., 2007; Harris and Šepelák, 2018), or of Al and Si in aluminosilicates (Mackenzie et al., 2000). These mesophases can accompany the step-wize disordering, amorphization, and overall structural transformations in solids exposed to mechanical action.

**FIGURE 3:**
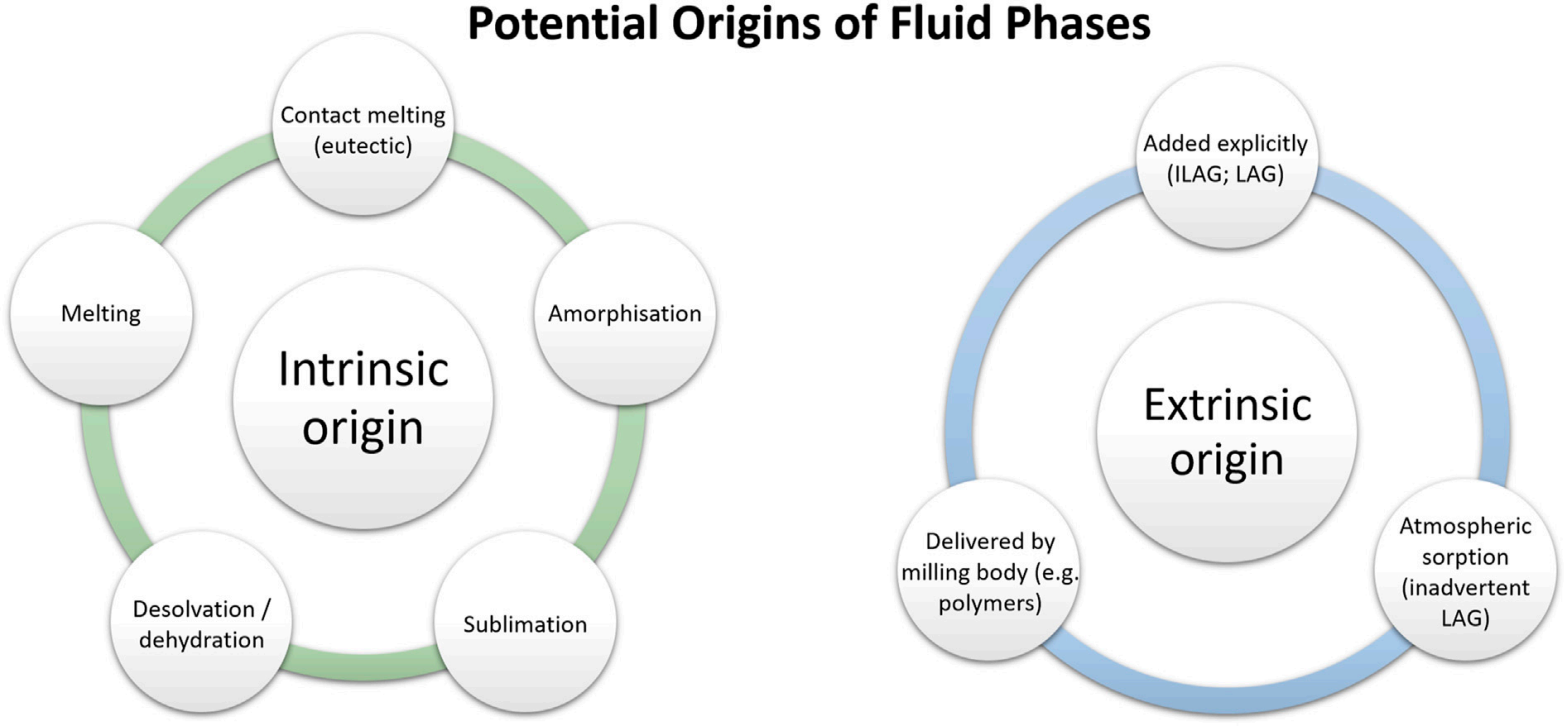
The potential origins of fluid phases in mechanochemical systems can be broadly classified as intrinsic and extrinsic, depending on whether they initiate within the solid phases or not.

Why then is the term *mechano*chemistry gaining more and more popularity? An intrinsic problem with applying the term *tribo*chemistry in the specific sense as originally proposed—as opposed to the previously existing term *mechano*chemistry—is that we must know the mechanism of the process. Specifically, the term tribochemistry should only be used if it is truly a solid-state reaction. This is not obvious, especially for organic compounds, even if we *start* with solids (Rothenberg et al., 2001; Tumanov et al., 2017). In many cases, the transformation itself, including chemical synthesis, likely occurs in a fluid phase; the possible origin of this fluid phase can be diverse (Figure 3). This is quite often the case for ball milling, grinding in a mortar, or processing in an extruder a mixture of solid organic compounds (Boldyreva, 2013). Generally, the origin of this fluid phase can be classified as being intrinsic or extrinsic to the reacting system itself. Intrinsic origins include melting (or contact melting) (Saratovkin and Savintcev, 1941; Gerasimov and Boldyrev, 1996; Urakaev and Boldyrev, 2000a; Boldyrev and Tkáčová, 2000; Chadwick et al., 2007; Michalchuk et al., 2014; Humphry-Baker et al., 2016; Fandiño et al., 2020; Haneef and Chadha, 2020), sublimation of solids (Kuroda et al., 2004; Mikhailenko et al., 2004), or dehydration/desolvation (Losev and Boldyreva, 2014) which result from the excess heating of mechanical impacts or bulk heating during mechanical treatment. Where a solids’ glass transition temperature is above the milling temperature, one can consider also the formation of transient amorphous phases (Descamps and Willart, 2016a). It has become very common to explicitly add liquid to a powder mixture to facilitate mechanochemical transformations, a process dubbed liquid assisted grinding (LAG) (Bowmaker et al., 2009; Bowmaker, 2013; Sarmah et al., 2019). This is an obvious extrinsic origin of a fluid phase. Even where researchers do not explicitly add liquid, the powder may “grab” liquid from the environment in a process dubbed inadvertent liquid assisted grinding (IA-LAG) (Tumanov et al., 2017).

Although the exact role of fluid phases in reactions that are assumed to occur in the solid state is not yet fully understood, various roles can be considered. The added fluid phase can certainly influence the mobility of material (by improving rheology, or by completely transferring the process into a solution or a melt) (Boldyreva, 2013; Lapshin et al., 2021). Additional to its influence on material mobility, melting can also drive erosion in microparticle impact (Hassani-Gangaraj et al., 2018), or can hinder impact-induced adhesion (Hassani-Gangaraj et al., 2017; Lapshin et al., 2021). A fluid, irrespective of its origin, can create hydrothermal conditions (Temuujin et al., 1998a; Boldyrev, 2002), can modify the mechanical properties of the solids *via* the Ioffe (Ioffe, 1936), Roscoe (Roscoe, 1934), or Rehbinder (Rehbinder, 1947) effects (*i.e.* the altering of bulk mechanical properties through surface modification) (Zhang et al., 2020; Dang et al., 2021), may influence triboelectric phenomena (Matsusaka et al., 2010), and can alter the relative stability of product phases through the selective stabilization of surfaces (Belenguer et al., 2018; Belenguer et al., 2019a). In many of these cases the process can and should be classified as tribochemical. However, where the process in fact occurs in the fluid phase, it can be no longer classified as tribochemical, nor does it unambiguously qualify to be denoted as a “dry” mechanochemical reaction.

By considering the possible intrinsic origins of fluid phases it becomes clear that some compounds are more likely to give rise to tribochemical transformations. For example, solids with high melting temperatures (primarily inorganic solids) will not melt or sublime during ball milling and therefore will most likely react tribochemically. In contrast, materials with low melting temperatures (*e.g.* most organic or coordination compounds) which are likely to melt during or as a result of mechanical treatment cannot be even strictly classified as mechanochemical, though tribochemical effects (*e.g.* electrostatic charging) may still be of importance at the elementary (molecular) level (Bowden and Gurton, 1949; Kajdas, 2013). This represents a critical difference between inorganic and organic “solid state” transformations under mechanochemical action (Boldyreva, 2013).

It follows from the above discussion that a reaction in a mechanochemical reactor that starts with solid reactants may not in fact be mechanochemical or tribochemical at all. This is the case when the chemical or physical transformation itself is not related directly to the absorption of the mechanical energy input. For volatile compounds, the solid reactants do not even require physical contact and can remain separated in space (Kuroda et al., 2004). In this particular case, the reaction is, strictly speaking, neither mechano-, nor tribochemical. In most reactions, however, no visible transformation is observed unless the compounds are treated in a mechanical apparatus. Yet, in many such cases the reaction is not mechanochemical, but thermal in nature; the role of the mechanical processing is limited to facilitating the mobility of the solid reactants, bringing them into contact with each other and/or by removing the solid products which are formed at the surface of the powder particles. Such processes are largely responsible for the success of new mechanochemical reactors such as the Resonant Acoustic Mixer (RAM) (Anderson et al., 2014; Michalchuk et al., 2018a; Titi et al., 2020). One could term such reactions as “mixing assisted thermal reactions”, as opposed to mechanochemical reactions. This can be taken as an analogue of stirred solution-phase reactions, wherein stirring does not *cause* the reaction, but simply facilitates the thermal reaction by driving mass transport.

These “mixing assisted thermal reactions” differ from “classical” thermal reactions in a few critical ways. “Classical” thermal reactions in solid mixtures (e.g. in high temperature solid-state synthesis) involve the heating of pre-mixed powders, wherein the powder remains largely unperturbed during the reaction. In contrast, reactions of thermal origin that occur during mechanical treatment are accompanied by dynamically changing local compositions (e.g. from mixing) and fluctuations in particle stress regimes. The mixing can be accompanied by the reduction of particle size (comminution), or changes in their agglomeration state. Together, these phenomena of macroscopic motion of particles, comminution or agglomeration, and the potential for strain-induced dissolution of one phase into another (Vasil’ev et al., 2006; Vasil’ev et al., 2009; Vasil’ev and Lomaev, 2011; Vasil’ev, 2012; Butyagin, 1984; Butyagin, 2005) yields intimate mixing across lengths of scale. One must also keep in mind that many solid + solid reactions are exothermic, since no entropy is gained during the reaction. Correspondingly, if the mechanical treatment is itself accompanied by significant heat evolution, mechanically initiated self-sustaining thermal processes become possible (Takacs, 1998; Bakhshai et al., 2002; Takacs, 2002; Delogu et al., 2004b; Maglia et al., 2004; Delogu and Takacs, 2014; Humphry-Baker et al., 2016). It follows that when mechanical mixing results in a chemical transformation, it is not clear *a priori* if the mechanical treatment of particles themselves plays a significant role, or if the particles are “merely brought into contact”. Regardless, any “mixing assisted thermal reaction” will be to a large extent governed by macrokinetics, *i.e.* heat and mass transfer processes (Urakaev and Boldyrev, 2000a; Urakaev and Boldyrev, 2000b; Delogu et al., 2003; Lapshin et al., 2021).

This thermodynamic feature—that solid + solid reactions are exothermic—raises an interesting question: do all mechanochemical reactions in fact have thermal mechanisms? When mechanical energy (U) is exerted on a system, some of the energy is absorbed by the material (e.g. as work, w) and the rest is released as heat (q): U = q + w. Using this rule for the conservation of energy, the amount of energy absorbed by the mechanically treated powder has been approximated by measuring the bulk temperature of the reaction vessel (Butyagin, 1967). What energy is then responsible for the transformation, the work or the heat? It is in fact the answer to this very question that distinguishes a mechanochemical transformation from a thermal transformation. Where the absorbed mechanical energy is itself responsible for the transformation (*i.e.* a mechanochemical reaction), the surrounding temperature has only indirect influence on the reaction. Once the transformation has occurred, excess energy is emitted from the system also as heat. Where a new product forms, for example in a multi-phase reaction, this can often take the form of exothermic nucleation. Although proving whether a reaction is thermo or mechanochemical can be challenging, a number of clear examples can be given, including the dark dimerization of aromatic compounds at high pressures (Engelke and Blais, 1994; Politov et al., 2010; Friedrich et al., 2020), the classic examples of the decomposition of metal halides by Carey Lea (Carey-Lea, 1892; Carey-Lea, 1894), the decomposition of alkali metal nitrates, bromates, and chlorates, which give different products and follows inverted trends upon heating and mechanical action (Boldyrev, 1972; Boldyrev et al., 1972; Urakaev et al., 1977; Boldyrev and Heinicke, 1979), or oxidation of gold by carbon dioxide (Thiessen et al., 1970). The relations between mechanochemical and thermochemical nature of the reactions can be compared with a situation when light and temperature act on a sample simultaneously. One can imagine a thermochemical reaction conducted in the presence of light: the mechanism does not become photochemical simply because light is present, it remains thermochemical. On the other hand, during a photochemical reaction, wherein the photon is absorbed, the reaction occurs on an excited state potential energy surface, and following relaxation, excess vibrational energy is emitted as heat. The heat is simply a byproduct of the photochemical transformation; one would not call such a reaction thermochemical.

It is therefore clear that many so-called “mechanochemical reactions” are not encompassed by the current IUPAC definition, which states that a mechanochemical process is “a chemical reaction that is induced by the direct absorption of mechanical energy” (Mechano-Chemical Reaction, 2009). Such distinctions, although semantic, play an important role when considering the types of physical phenomena which may play a role in driving the observed reaction. For example, effects of adiabatic compression, or the generation of vibronically excited states are unlikely to play a role in “mixing-assisted thermal reactions.” On the other hand, slow nucleation and crystal growth—which presumably dominate such thermal reactions—are very different from the fast cooperative interfacial propagation processes which can be expected for “true” mechanochemical reactions in which mechanical energy is directly transferred into high-level vibrational or electronic excitations (Coffey and Toton, 1982; Dlott and Fayer, 1990; Tokmakoff et al., 1993; Luty et al., 2002; Eckhardt, 2006; Michalchuk et al., 2018b; Michalchuk et al., 2019a; Michalchuk et al., 2021).

Selecting a proper term for a process involving solids that occurs in response to mechanical action is thus intrinsically challenging; the correct term can be only given after the mechanism for the reaction has been established. Even for fluids, deciding on the term “tribochemistry” or “mechanochemistry” may be not always straightforward. Not only can mechanical treatment convert solids to fluids, but it can also convert fluids to solids. Shearing of fluids (*e.g*. lubricants) often yields rigid supramolecular structures, which can inversely affect fluid properties such as viscosity (Apóstolo et al., 2019). Compression of fluids ultimately leads to their solidification. The solidification of long chain alkanes at relatively low pressures imposes serious implications for their lubricating effects. In certain examples such as mixtures of squalene with poly-α-olefins, the solidification at *ca.* 1 GPa is not associated with crystallization, but rather with the formation of a glassy state with reduced mobility. Other examples are known, such as with 1-dodecanol, wherein moderate pressures induce crystallization of polymorphic modifications which favor sliding, and hence increase their lubricating ability: superlubricity (Reddyhoff et al., 2021). Finally, as in 1:1 mixtures of pentane:isopentane (Liu and Pulham, 2020), the pressure-induced solidification of fluids can be also selective and cause only a specific component to crystallize. As a result of this selective crystallization, the composition of the fluid (in fact, a mesophase) changes. These examples serve to show just how interwoven the *tribo*- and *mechano*-sciences really are, and indeed the importance of understanding their connectivity.

This vague borderline between “solid-state” and “fluid-state” processes under mechanical action only exacerbates the problem of defining the correct terminology to refer to a process involving mechanical action. Moreover, there is significant likelihood that many reactions will need to be reclassified as our understanding of the mechanisms of mechanochemical reactions expands. To paraphrase N. Copernicus, *we know what we know, but we do not yet know what we do not know*. This reality is of course impractical and indeed superfluous for most researchers who are more interested in the outcome of mechanical treatment rather than in the detailed mechanism of the transformation. What *is* important, however, is that the process can be reproduced by others based on the original, recorded description. This requires effective and accurate communication of the mechanochemical protocol used, with meaningful descriptions of all the parameters that are known to influence mechanochemical reactions. Hence, although we cannot know *a priori* how a seemingly “solid + solid” reaction will occur, we can know for sure how we treat the sample and analyze the outcome. It is also important to determine and record the appearance and state of the sample at the start and end of our treatment. It is this information rather than a name itself that must be reported and controlled as carefully as possible. Only in such a way can we hope to identify under what conditions solids react, and how to implement this technology most effectively.

## The Breadth of Mechanochemistry

If we define a reaction as thermochemical, it is sufficient to indicate the temperature at which it occurs. If the reaction is not isothermal, a protocol of modifying the temperature with time is required. For a photochemical reaction, energy, polarization, the intensity of light, the spatial characteristics of the irradiation (uniform, local, one-sided, etc.) must be defined. In contrast, where irradiation is discontinuous, the duration and frequency of light pulses must be stated.

**FIGURE 4:**
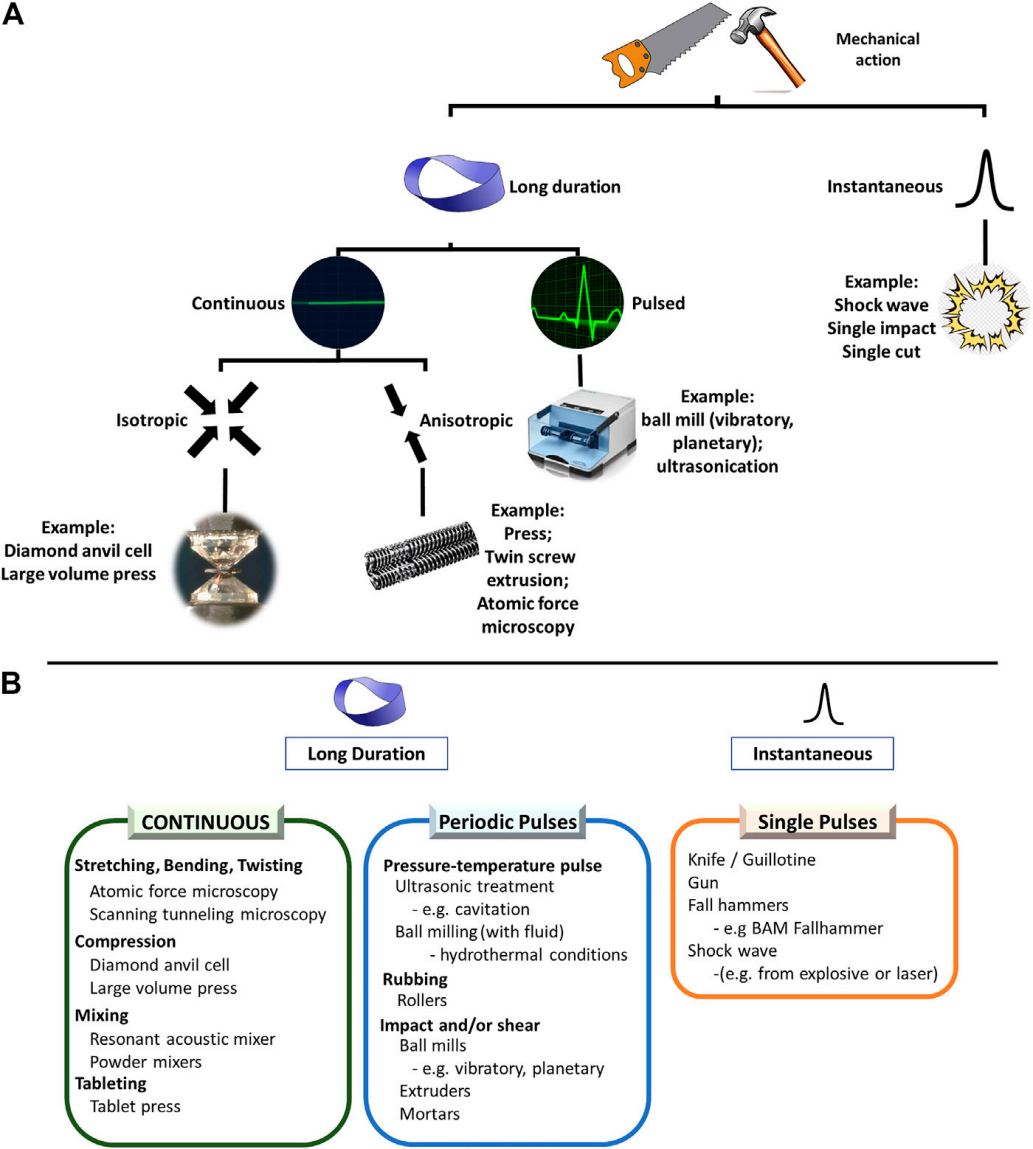
Representation of the breadth of mechanical actions which are frequently used in mechanochemistry. **(A)** A broad classification of the types of mechanical action encountered in mechanochemical reactions. **(B)** Examples of common devices under each category.

For a mechanochemical transformation the type of mechanical action, experimental conditions, the composition and appearance of samples are much broader than for a thermal, or a photochemical reaction (Figure 4). In fact, the very questions as to “how we treat a sample” and “in what state the reactant and product phases exist” are not easily defined. Increasingly, new features are being identified to be crucial for determining the reaction pathway of mechanochemical transformations. For example, the ability for starting reagents to exist in different solid forms (polymorphs, polyamorphs, particles of different size and shape) is unique to the solid state. However, the starting forms of the solid reactants are often not reported in literature, under the assumption that they will be modified by treatment anyway. However, a starting polymorph can play a non-negligible role in determining the outcome of a mechanochemical transformation (Bouvart et al., 2018). Evidence has suggested also that the particle sizes of reagent materials can influence the mechanochemical transformation (Michalchuk et al., 2017). Similarly, the presence of unobservable contaminants (seeds) (Fischer et al., 2016) or crystal defects (Boldyrev et al., 1979) can be critical to the success or failure of a mechanochemical transformation. It is without doubt that more influential parameters will become known with further studies of mechanochemical reactions. At present we can only make every effort to record and describe as many parameters as possible regarding mechanochemical transformations and remain ready to adapt to new developments as they emerge.

The type of mechanical action used to promote mechanochemical transformations has a clear influence on its outcome (Michalchuk et al., 2013; Tumanov et al., 2014). This has become increasingly important owing to the rapidly expanding repertoire of mechanochemical reactors. These range from the original mortar and pestle and ball milling (e.g. planetary and vibratory) to twin screw extrusion (TSE), Resonant Acoustic Mixing (RAM), diamond anvil cell (DAC) technologies, and atomic force microscopy (AFM). The choice of mechanochemical reactor has significant influence on *how* the stress is applied to the sample in the first place (Figure 4).

**FIGURE 5:**
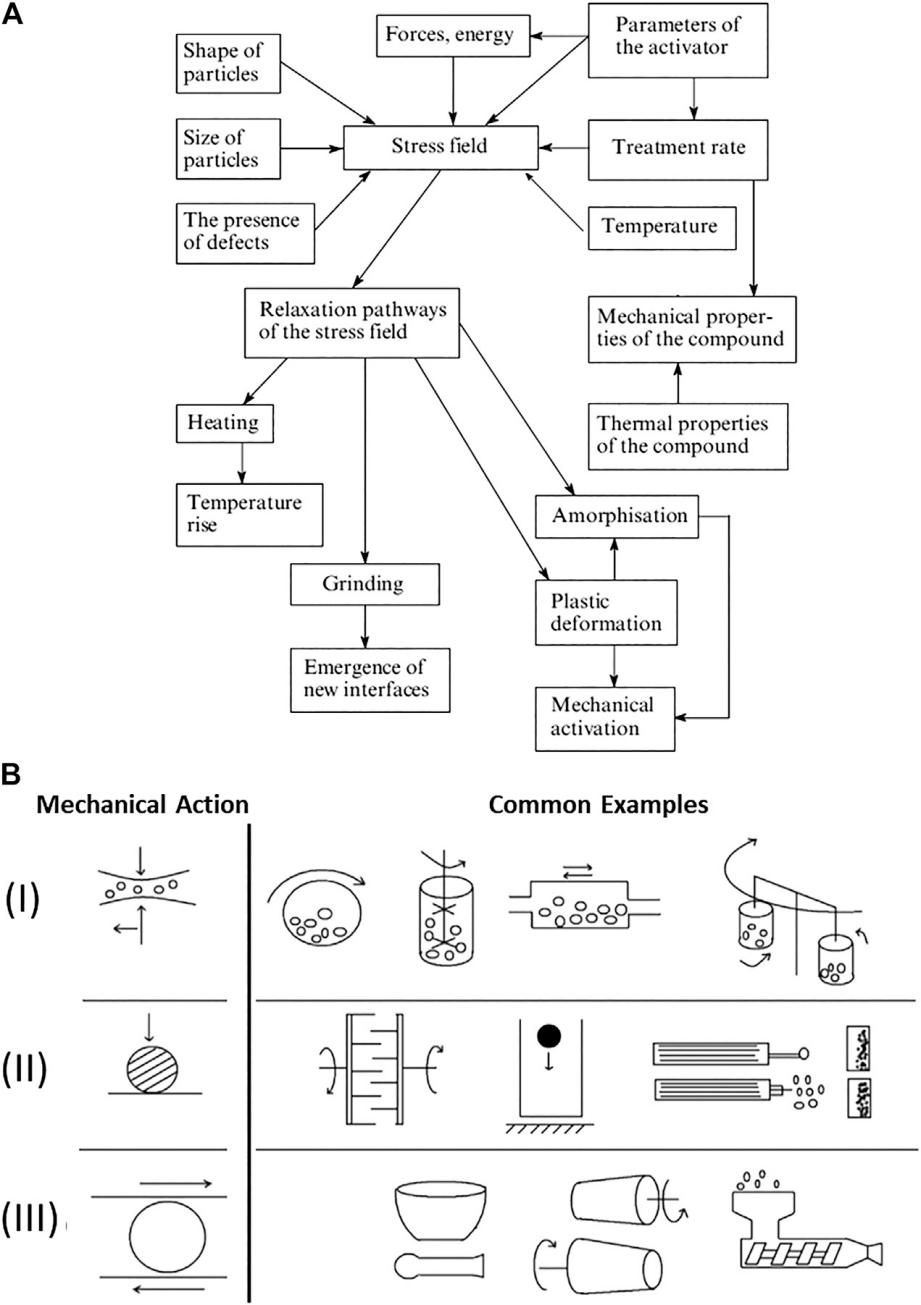
Different influence of mechanical action on solids. **(A)** Flow diagram for the formation and subsequent relaxation of stress which are involved in mechanochemical reactions in solids, adapted from Ref (Boldyrev, 2006) **(B)** Schematic representation of the breadth of mechanical actions experienced in different types of mechanochemical reactors. (I) Combination of impact with shear/friction in various types of ball mills and attritors: (from left to right) rotational mill, attritor-stirring ball mill, vibration mill and planetary mill). (II) Dominated by impact in: (from **left to right**) pin mill, fall hammer, jet mill. (III) Dominated by shear/friction: (from **left to right**) mortar and pestle, rolling mill, and extruder. Figure adapted from Ref (Lapshin et al., 2021).

The temporal evolution of mechanical action can be regarded as a first parameter to consider when distinguishing mechanochemical regimes of treatment. If a shock wave or a single pulse (e.g. a drop hammer, a knife, a gun, or a jet mill) is used, there is a single excitation event, followed by relaxation of the material *via* various channels (Figure 5A) (Boldyrev, 2006) For example, this is important in the case of the mechanochemistry of explosives (Michalchuk et al., 2019a), wherein the mechanical impact is suggested to induce super-heating of lattice phonons (Dlott, 1999), which ultimately yields a chemical response *via* dynamical metallization (Michalchuk et al., 2018b). Chemical transformations at a crack tip fall also into this category. As the crack propagates through the material, the exceptional transient stresses generated at the crack tip induce highly metastable states which, after the crack dissipates, relax through chemical recombination (Gilman, 1960; Gilman and Tong, 1971; Gilman, 1996; Gilman, 1998; Urakaev, 2007; Urakaev, 2008). During explosive initiation and detonation, chemical changes may in fact precede heat, such that the process is athermal and thus truly mechanochemical (Gilman and During, 2006). The rate of crack propagation (akin to the magnitude of a shock wave in explosives) dictates the magnitude of metastability and can even influence the relaxation product, *i.e.* the chemical products of the mechanochemical decomposition (Urakaev et al., 1977; Urakaev and Boldyrev, 2000).

Typically, treatment of a sample by a single mechanical pulse is used in model studies, when the details of the effect of the mechanical action on the sample are studied—light, electron, radicals, or gas emission, heat evolution, propagation of the deformation wave, generation of phonons, a hot spot formation, etc. For example, the effect of controlled single impacts on the decomposition of silver oxalate to form Ag nanoparticles has been recently investigated by Delogu and colleagues (Torre et al., 2020). Studies on single pulses are particularly well suited for the *in situ* study of fast transformations, and indeed form the basis of safety technologies e.g. in testing explosives (*c.f.* the BAM Fall Hammer or the Rotter Impact Device).

If the mechanical action is long-lived, however, the relaxation event is altered, and the complexity of the problem increases. **Long-lived** action can itself be divided as being **continuous or pulsed**. In the former, the stress is applied and maintained. The stress must relax on a so-called force-modified potential energy surface (FM-PES), which can be significantly different than the surface of the fully relaxed material. Moreover, the rate at which the stress is applied can influence on the effective FM-PES upon which relaxation occurs (Fisch et al., 2015). Atomic force microscopy (AFM) can be used to stretch continuously a selected bond in a molecule. AFM and other dedicated stretching or bending device can be also used to deform a macroscopic object—a single crystal, a polycrystalline sample, a polymeric fiber—elastically or plastically. It can be used to study physical and chemical transformations in such strained samples (Boldyreva and Sidelnikov, 1987; Gutman, 1998; Rusanov et al., 2004; Chizhik et al., 2018). Alternatively, one can subject a sample to hydrostatic compression (a small amount in a diamond anvil cell, or a larger amount in a large volume press) and study either the transformations induced by compression itself, or the effect of compression on thermal, or photochemical transformations (Zakharov and Boldyreva, 2019; Galica et al., 2020).

Most devices are based on repeated mechanical treatment of samples in the form of pulses. In the case of **repetitive dynamic stressing**, relaxation of the stress occurs on the unstressed PES. However, depending on the intensity, pulse shape, duration and frequency of pulses, the relaxation may not be complete. This offers a potential means to accumulate energy with successive pulses (Butyagin, 1971; Belenguer et al., 2019b). Additionally, the relaxation channel (Figure 5A) can change during the course of the reaction, for example as the result of reduction in particle size toward the grinding limit (Boldyrev et al., 1996). The rate of pulsing can vary significantly, from *e.g.* 30 Hz in a conventional ball mill to tens of kHz using ultrasound radiation (Zhou et al., 2020). The types of mechanical action can be also different, including impact, shear stress, friction, rubbing, and cleavage, or any combination thereof, Figure 5B. Moreover, different types of action can be applied to fractions of the sample located at different sites within the same milling jar (Michalchuk et al., 2013), or can vary between successive pulses. The type and variability of mechanical action does not depend only on the choice of the mechanoreactor, but also on 1) the protocol of operation for the mechanochemical reactor (Zyryanov, 2008), 2) the nature of the sample (e.g. its rheology) (Hutchings et al., 2017; Michalchuk et al., 2017), 3) the presence of additives (e.g. liquids, polymers), and 4) the nature the milling bodies (Michalchuk et al., 2019b) (e.g. their material, mass, density, size and the total number). Each new pulse can interact with the same particle in potentially different geometry, or with another particle altogether. Under such dynamic stressing conditions, little is understood about what mechanical processes actually occur, and even less is understood about how to control them. Customized mechanochemical reactors which mimic specific types of mechanical action have been constructed in attempts to minimize the variation between reaction sites and successive impulses, and to monitor step-by-step the evolution of systems under consistent and repetitive stress (Tumanov et al., 2011; Tumanov et al., 2014). By using such customized mechanochemical reactors it has been possible to attempt to rationalize the outcome of more complex commercial mechanochemical reactors, such as ball mills or attritors (Michalchuk et al., 2013; Michalchuk et al., 2014). It is likely that at different stages (comminution, mixing, generating defects, reaction itself) effective mechanochemical transformations require that a sample be processed in different machines which are optimized for these particular phenomena. Developing customized mechanochemical reactors to simulate and study systematically mechanochemical transformations under idealized mechanical action is exceptionally important to rationalize how and why mechanical treatment exerts its effects. To this end, new mechanochemical reactors capable of probing at the nanoscopic level the evolution of solids exposed to mechanical treatment are being also envisioned (Thiessen, 2016).

Identifying the type of mechanical regime, and indeed the type of mechanical action is not obvious but in exceptional circumstances. In the general case, no sharp boundaries exist between the regimes of mechanical treatment. For example, in ball milling experiments the powder is constantly relocating through the jar. Consequently, the frequency and intensity of pulsed loadings is not constant. Indeed, an apparently “repetitive dynamic” regime may be in fact more accurately described by “single pulse” regime of reactivity. In contrast, if powder is tableted during impact, material remains under some compression. Moreover, when tableted, both the type of mechanical impact (e.g. from free impacts to restricted impacts) or potential for shear (e.g. particles against the jar walls) are greatly affected. Hence, when tableting occurs the regime of mechanical treatment changes unexpectedly. The stochastic and time-dependent formation of “reactive” and “unreactive” contacts, which are affected by mixing, can be also unpredictable. For example, in a two-phase reaction, only mechanical treatment of contacts which involve both phases (*i.e.* a heterogeneous contact) will react. In contrast, homogeneous contacts will not undergo reaction. Thus, the relaxation of mechanical stress depends also on the structure of the inter-particle contacts, which vary unpredictably with time. Even if one starts with treating a single phase (for example, when a polymorphic transition, an amorphization, or a decomposition are studied), as soon as the product starts being formed, the system becomes at least “binary”, or may contain an even larger number of unique phases. Correspondingly, each successive impact may strike already reacted material, rather than the remaining unreacted particles. This has dramatic consequences on the kinetics of the transformation (Michalchuk et al., 2018c). Even such “single phase” mechanochemical transformations turn out to be strongly controlled by macrokinetics, i.e., mass and heat transfer, comminution, mixing and aggregation (Delogu et al., 2003).

## A Need for More?

A true designation of a mechanochemical (or tribochemical) reaction requires a thorough understanding of its mechanism. In all but a few cases, this understanding is far from being achieved (*The Breadth of Mechanochemistry*). We must therefore ask the question: Is an understanding of the mechanism a prerequisite to garnering control over potentially mechanochemical reactions at all? We might not need to know what *exactly* is occurring, but we *must* understand collectively what actions are being done, and how to describe these actions amongst the community. The strategies being used across the domains of mechanochemical reactions must be effectively communicated. Only in this case can the community pool knowledge and ultimately achieve the mechanistic understanding required to develop robust and meaningful *names*. This need to develop a common language in reporting science has been crucial for example in progressing the field of crystallography. The creation of a *Crystallographic Information File* (CIF) (Hall et al., 1991) facilitated the deposition and automatic processing, visualization and validation of crystallographic structural data. Additionally, the CIF format facilitated the wide adoption of common standards for good practices in the collection and processing of structural data by highlighting to researchers which experimental parameters must be controlled and reported, such that anyone can validate and reproduce the results (Brown and McMahon, 2002; Brown and McMahon, 2006; Vaitkus et al., 2021). During its inception, researchers understood that the field remained in development, both in terms of technology and in understanding which parameters were required for the effective communication of crystallographic data. Hence, the CIF file was established to be dynamic and “naturally evolving”. The process continues still, including increasingly difficult cases such as structural data from powder diffraction, for modulated structures, and only very recently expanding to include high-pressure data (Dziubek, 2020). This situation is very similar to that which faces mechanochemistry today.

It is certain that no universal set of parameters or nomenclature can span the entirety of mechanochemical research. The diversity in mechanical action, stressing regimes, phases present in the system as reactants, additives, reactor materials, or inadvertently penetrating water or impurities, to list just a few parameters that need to be controlled, are too diverse. Yet, this is again synonymous with crystallography, wherein different diffractometer set-ups, collection strategies, sample types, all require a specific set of parameters to be recorded. It is instead likely that effective communication of mechanochemical sciences must evolve to include as many standard parameters (e.g. additives and reactant composition) as possible, whilst allowing the flexibility to dictate mechano-reactor specific information.

**FIGURE 6:**
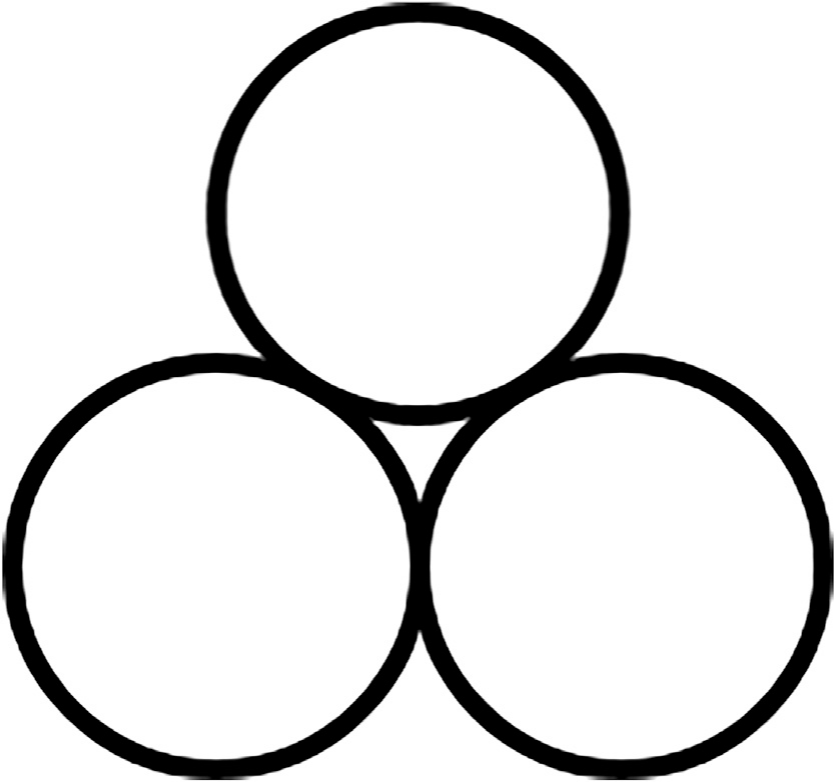
Symbol proposed for mechanochemical activation by Hanusa *et al.* (Rightmire and Hanusa, 2016).

A significant step in this direction to standardize nomenclature is credited to Hanusa *et al.*, Figure 6 (Rightmire and Hanusa, 2016). The addition of energy into chemical reaction schemes is easily denoted for traditional methods. For example, the photochemical activation is denoted by ℏω, describing the addition of a quantum of light. Similarly, heating is denoted by Δ. The “three-balls” symbol was suggested to be used to denote any mechanochemical transformation (Rightmire and Hanusa, 2016), and the simplicity of this symbol has thus far rendered it popular amongst a significant part of the organic mechanochemical community.

This symbol represents an excellent step toward realizing the need to discern clearly transformations resulting from mechanical treatment from those initiated by thermal, photo, or electrochemical stimuli. However, despite its popularity, the “three ball” symbol appears to have drawbacks when it is supposed to be used as a universal denotation for mechanically induced reactions.

Culturally, the symbol coincides with that of an ancient religious symbol that has existed since prehistory and is found throughout the world. A nearly identical symbol was subsequently adopted as the symbol for the “Banner of Peace”, symbolizing the protection of cultural property in times of war (Roerich Pact, 1935).

As was illustrated in the previous Sections, mechanochemistry differs from other branches of chemical science in that there exist many ways in which the mechanical action can be applied to the system. These different mechanical actions frequently yield different results. Material treated in a vibratory ball mill will be subjected to very different mechanical forces than the same powder treated in a planetary ball mill. Treatment in an extruder, jet mill, or in the rolling mill will be even more different. A single symbol cannot be therefore sufficient to define a general regime of mechanical treatment. Moreover, the presentation of milling balls is not relevant for example in many organic syntheses wherein excellent and scalable results were obtained not by impact in ball mills, but by shear in mortars, extruders, or rollers (Crawford and Casaban, 2016; Andersen and Mack, 2018b; Stolar et al., 2019; Ali El-Remaily et al., 2020; Crawford et al., 2020; Margetić and Štrukil, 2020). In fact, a vast majority of efficient and scalable mechanochemical types of treatment are “ball free” (ultrasonic, RAM, TSE, jet milling, pin milling, rolling-milling, grinding in a mortar, to name just a few).

### The Need to Specify Mechanical Action

The specific reactivity of materials under different types of mechanical action has been known for decades. Most famous, perhaps, is the reactivity of energetic materials, whose friction and impact sensitivities can differ enormously. Correspondingly, specialists in energetic materials research make explicit effort to ensure that the type of mechanical action is reported alongside the magnitude of perturbing energy; this reporting and testing procedure has been internationally standardised (UNECE, 2009). It is not surprising to find that the behavior of other organic systems is also mechanoreactor-dependent. For example, in the case of the “piroxicam + succinic acid” system, a co-crystal is formed from the two components on repetitive impacts by a falling ball (Tumanov et al., 2014). On the contrary, piroxicam: succinic acid co-crystal decomposes into the components on grinding when friction dominates (Tumanov et al., 2014). Uninterrupted ball milling of a mixture of glycine and malonic acid gives a different product as compared with the outcome of a treatment that is interrupted intermittently and where the sample is mixed manually (Michalchuk et al., 2013; Michalchuk et al., 2014). The work by Belenguer and colleagues demonstrates clearly how the selective choice of ball milling protocol can be used to target with great reproducibility the polymorphic form of organic solids (Belenguer et al., 2018; Belenguer et al., 2019a). The epsilon-polymorph of chlorpropamide can be transformed into the alfa-polymorph only on cryogrinding at the liquid nitrogen temperature, whereas no transformation is observed on ball-milling at room temperature (Drebushchak et al., 2011). Mechanical treatment of the beta-, gamma-, delta-polymorphs of chlorpropamide gives different products, depending on the choice of the starting form, the type of treatment (grinding in a mortar, simulated impact and shear, or during ball milling) and the presence of a small liquid additive (Bouvart et al., 2018). Liquid assisted Resonant Acoustic Mixing of carbamazepine and nicotinamide gives different products depending on the magnitude of acceleration (Michalchuk et al., 2018a). Such examples are not limited to organic reactions. For example, depending on the rate of crack propagation in crystalline nitrates and chlorates, different products are formed on their mechanolysis (i.e. mechanochemical bond rupture and formation of radicals) (Urakaev et al., 1977). The dissolution rate of quartz in hydrofluoric acid was also shown to depend critically on the type of mechanical treatment, with vibratory milling yielding slower dissolution kinetics than jet milling (Heinicke, 1984). Similar effects are observed for the formation of a Zn—fumarate metal-organic framework, which forms the tetrahydrate upon intense treatment in a SPEX-8000 mill, but a pentahydrate upon restricted impact treatment or laboratory vibratory ball milling (Strobridge et al., 2010; Tumanov et al., 2012). The rate of mechanochemical synthesis of inorganic solids has been also shown to depend critically on the choice of mechanochemical reactor. For the synthesis of aluminosilicate geopolymers from 1:1 layer-lattice minerals, planetary ball milling at 400 rpm using a sample: milling media mass ratio of 1:50 (3 mm diameter ZrO_2_ balls) took 20 h of mechanical treatment. Instead, this reaction took only 15 min using a more energetic vibratory ball mill with a tungsten carbide bowl and rings (MacKenzie et al., 2007).

### A Picture is Worth a Thousand Words: Pictographic Representations of Mechanochemistry

It is thus increasingly clear that many often-over-looked parameters can influence on a mechanochemical transformation. Additional to the choice of mechanoreactor (Tumanov et al., 2014), this includes the initial polymorphic form (Bouvart et al., 2018), the inclusion of additives (solid, liquid, or gaseous) (Fischer et al., 2016; Hasa et al., 2016; Belenguer et al., 2018), the bulk temperature of the mechanoreactor (Andersen and Mack, 2018a), the atmosphere in which the reaction occurs (Tumanov et al., 2017), the rate of mechanical stressing (Michalchuk et al., 2018a), the mass and material of the milling balls and jars (Kulla et al., 2019; Michalchuk et al., 2019b; Germann et al., 2020). Certainly, many additional and unsuspected parameters can also exert significant influence over control in mechanochemistry.

Despite this, modern mechanochemical literature typically describes the applied mechanical action in general terms. For example, transformations are often described as occurring under “ball milling conditions”; in many recent papers the mechanochemical reactions are denoted with a general symbol as in Figure 6. The onus thus remains on the careful reader to identify the type of mechanoreactor, and subsequently the main type of mechanical action being applied. This information can be given in a concise form in the Experimental, or, even more difficult to see immediately, in Supplementary Material. A considerable amount of crucial information is therefore not immediately visible, even if available in the publication. In the absence of a “check list” indicating all the important experimental details that *must* be reported, it is also not uncommon that crucial information is missing. This is often the case, when mechanochemical work is being reported and published in general interest chemistry, physical chemistry, synthetic organic chemistry journals, rather than in the specialized solid-state, materials science, chemical engineering, or tribochemistry journals.

**FIGURE 7:**
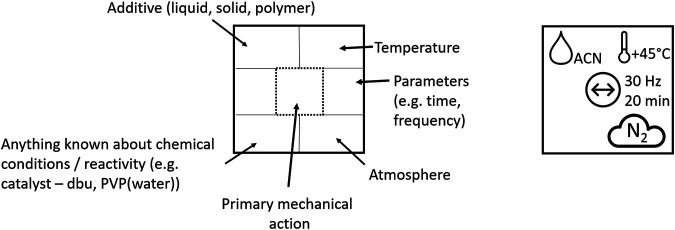
Mechanochemistry at a glance. **Left:** Schematic representation of the structure of the proposed mechanochemical reaction symbol. Types of symbols see Table 1. **Right:** Example of the symbolic representation of a mechanochemical reaction performed in a vibratory ball mill for 20 min at 30 Hz under LAG conditions using ACN at 45°C under N_2_ atmosphere.

We propose a demonstrative model for a more elaborate, albeit concise, description of a mechanochemical reaction. It is our hope that such a pictographic “nomenclature” will facilitate clarity of communication whilst encouraging researchers to share vital information regarding mechanochemical syntheses. Inspired by the standard elemental symbols used on the periodic table, the example nomenclature emphasis is on the type of mechanical action, which is placed at the center of the symbol. Additional parameters which are currently known to affect the outcome of mechanochemical transformations are indicated systematically in the surrounding space, Figure 7. Such a formalism could make great strides toward ensuring comparable, consistent, and reproducible reporting of mechanochemical reactions.

In this representation, parameters are given that are known to affect the results of a reaction. These include:

*Additive:* a solid, liquid, or polymer that is added to the mixture, in order to facilitate the physical transformation, and may or may not be chemically involved in the transformation. This includes processes such as liquid assisted grinding (LAG), ionic liquid assisted grinding (ILAG), polymer assisted grinding (POLAG), and the addition of polymer to affect rheology. We suggest that the symbol contains a short acronym to label the additive, for example acetonitrile—ACN.
*Temperature*: In cases where the vessel temperature is explicitly controlled (either heating or cooling), the temperature can be indicated here. We suggest that the temperature is denoted alongside the associated symbol.
*Known chemical reactant*: In addition to denoting the additive, any species which are known to be chemically involved in the reaction should be denoted. This includes catalysts, also in the cases where the milling bodies act as catalysts themselves. We suggest that a short acronym be added to label the reactant species, for example 1,8-Diazabicyclo [5.4.0] undec-7-ene—DBU.
*Atmosphere*: An indication as to the atmosphere within the milling vessel should also be specified. This may be ambient atmosphere (atm), dry air (dry), or any other atmospheric conditions.
*Primary mechanical action*: A symbol should be selected to best represent the type of mechanical action being implemented. Additional information regarding reaction time and mechanoreactor conditions (e.g. frequency) should also be given.

These suggestions extend significantly the idea that has been recently expressed in the literature that at least the catalyst which is important for a mechanocatalysis must be added to the symbol of the mechanical treatment (Pickhardt et al., 2020).

To facilitate the consistent use of this system, we have established a preliminary set of suggested symbols, Table 1. The set of symbols can be easily extended e.g. for new types of mechanoreactors or additives.

**TABLE 1.** Potential symbols to be used for each major type of mechanical action and experimental conditions.

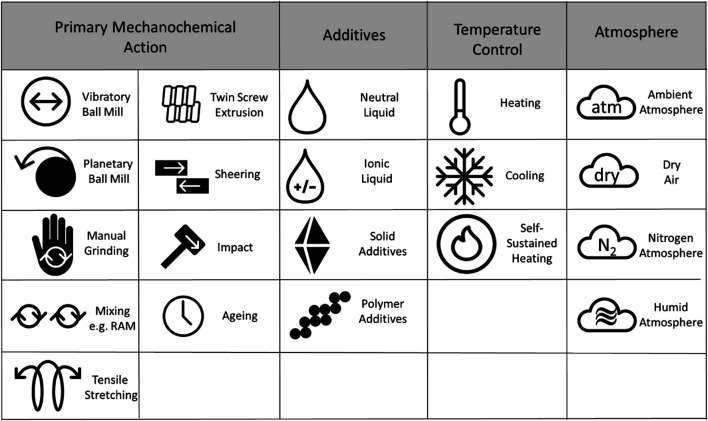

### Examples of Graphical Representations for Mechanochemistry

Let us consider a few examples to illustrate, how much clearer experimental conditions can be at a first glance if we use these pictographic descriptions.

**FIGURE 8:**
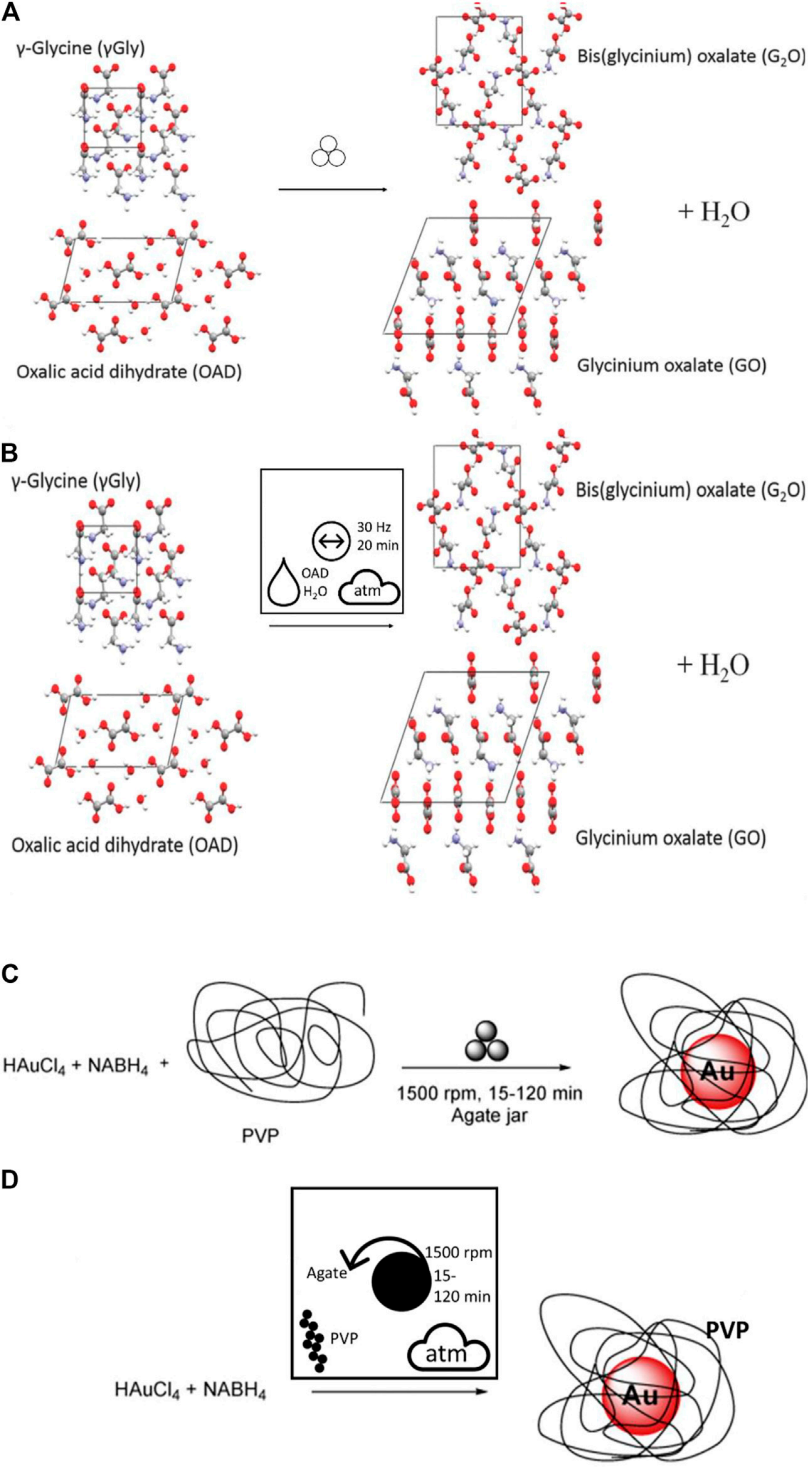
**(A, B)** Comparison of a simple vibratory ball milling co-crystal formation. **(A)** The original nomenclature (Michalchuk et al., 2017) and **(B)** the proposed nomenclature. **(C–D)** Comparison of nomenclature for the BUMS of Au nanoparticles by planetary ball milling with **(C)** original nomenclature and **(D)** recast using the proposed pictograms. Note the choice of planetary milling is only clear in the latter.

As a first example, we compare the frequently used mechanochemistry symbol (Figure 6) to the alternative nomenclature proposed here, with a simple ball mill grinding cocrystal formation, Figure 8 (Michalchuk et al., 2017). In the original nomenclature, no additional information is visible as to the experimental conditions. In contrast, the proposed new graphical nomenclature immediately allows the reader to identify this as a vibratory ball milling reaction at 30 Hz for 20 min, under atmospheric conditions. The LAG conditions are also visible in the new reaction, in which the water originates from the OAD. Similarly, examples of bottom-up mechanochemical synthesis (BUMS) of inorganic nanoparticle formation are being increasingly common (Rak et al., 2014; Rak et al., 2016; de Oliveira et al., 2020a; de Oliveira et al., 2020b; de Oliveira et al., 2020c). BUMS involve a number of critical processing features, including the addition of reducing agent and stabilizers alongside the mechanochemical conditions. The use of pictographic nomenclature makes immediately clear the use of planetary ball milling for BUMS of Au nanoparticles from HAuCl_4_, Figure 8B (Moores, 2018).

**FIGURE 9:**
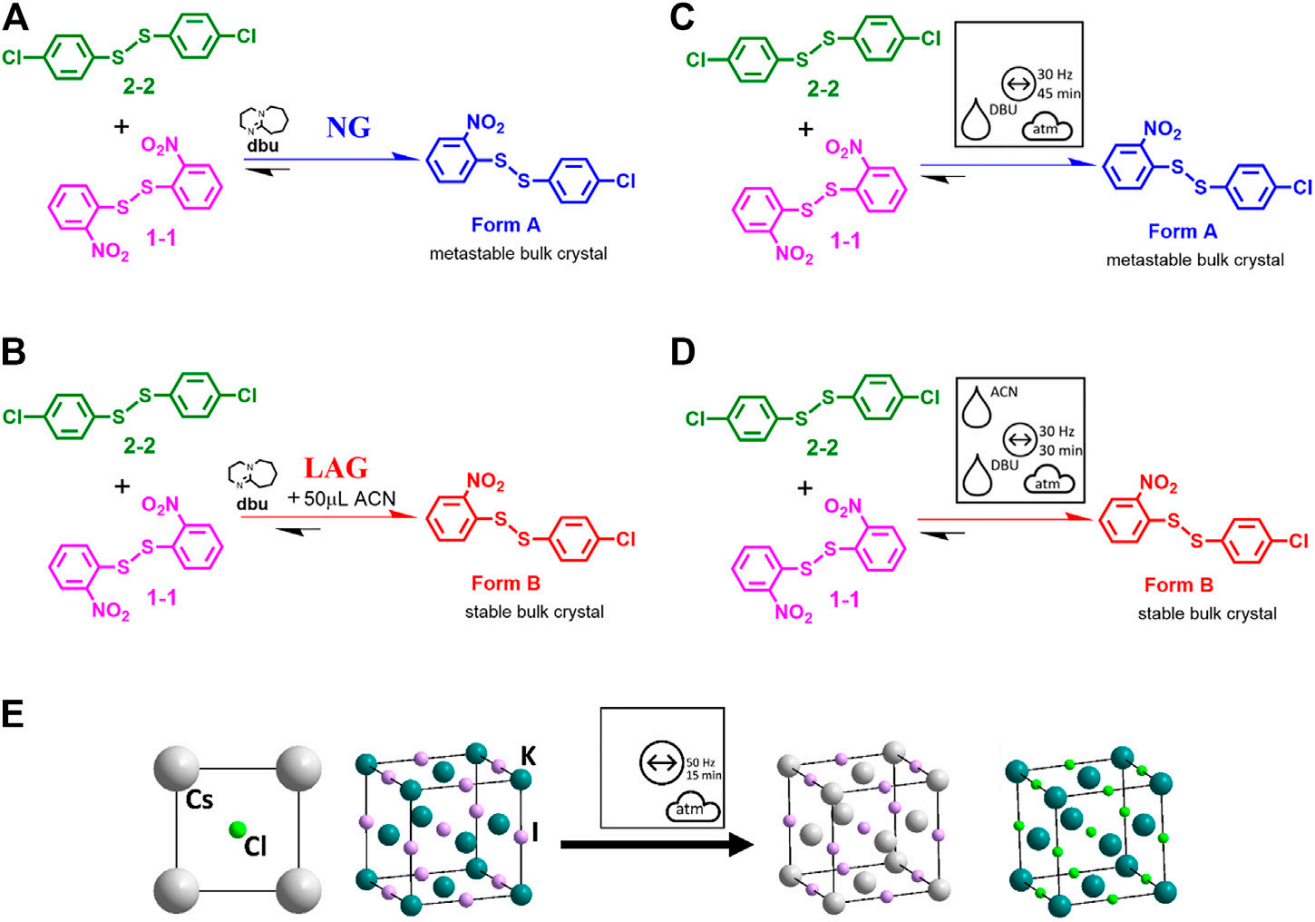
**(A–D)** Reaction schemes for the mechanochemical disulfide bond formation. **(A, B)** adapted from literature, Ref (Belenguer et al., 2019b). **(C, D)** The same reactions using the proposed general symbols for mechanochemistry. **(E)** Reaction schemes for ionic exchange in inorganic salts in a vibrational mill (Lampronti et al., 2021).

It is next worth considering a more complex reaction—that of the vibratory ball milling-induced disulfide exchange reaction, Figure 9 (Belenguer et al., 2019b). In the original publication, the ball milling reaction was denoted as in Figures 9A,B. The catalyst (here DBU) is shown above the reaction arrow, as well as the LAG conditions in ACN. No further information is given as to the nature of the energy source, the physical state of the dbu, the atmospheric conditions or any temperature control. Instead, the readers must find this information for themselves. All of this information is instead visible pictorially using the proposed general nomenclature for mechanochemical reactions, Figures 9C,D. It is immediately evident that the reaction is conducted by vibratory ball milling at 30 Hz under atmospheric conditions, over 30 min for LAG and 45 min for neat grinding (NG), and in the presence of liquid DBU. Furthermore, it is obvious that the two reactions differ only in the presence (or not) of the liquid additive, ACN. Hence, with little extra effort, an enormous amount of additional information (much of which was not present in the original manuscript) is now immediately accessible to the reader. This simplicity of the pictographic representation is equally applicable to inorganic reactions, such as the double salt metathesis between KI and CsCl under vibratory ball milling conditions, Figure 9E (Ban et al., 2017; Lampronti et al., 2021).

**FIGURE 10:**
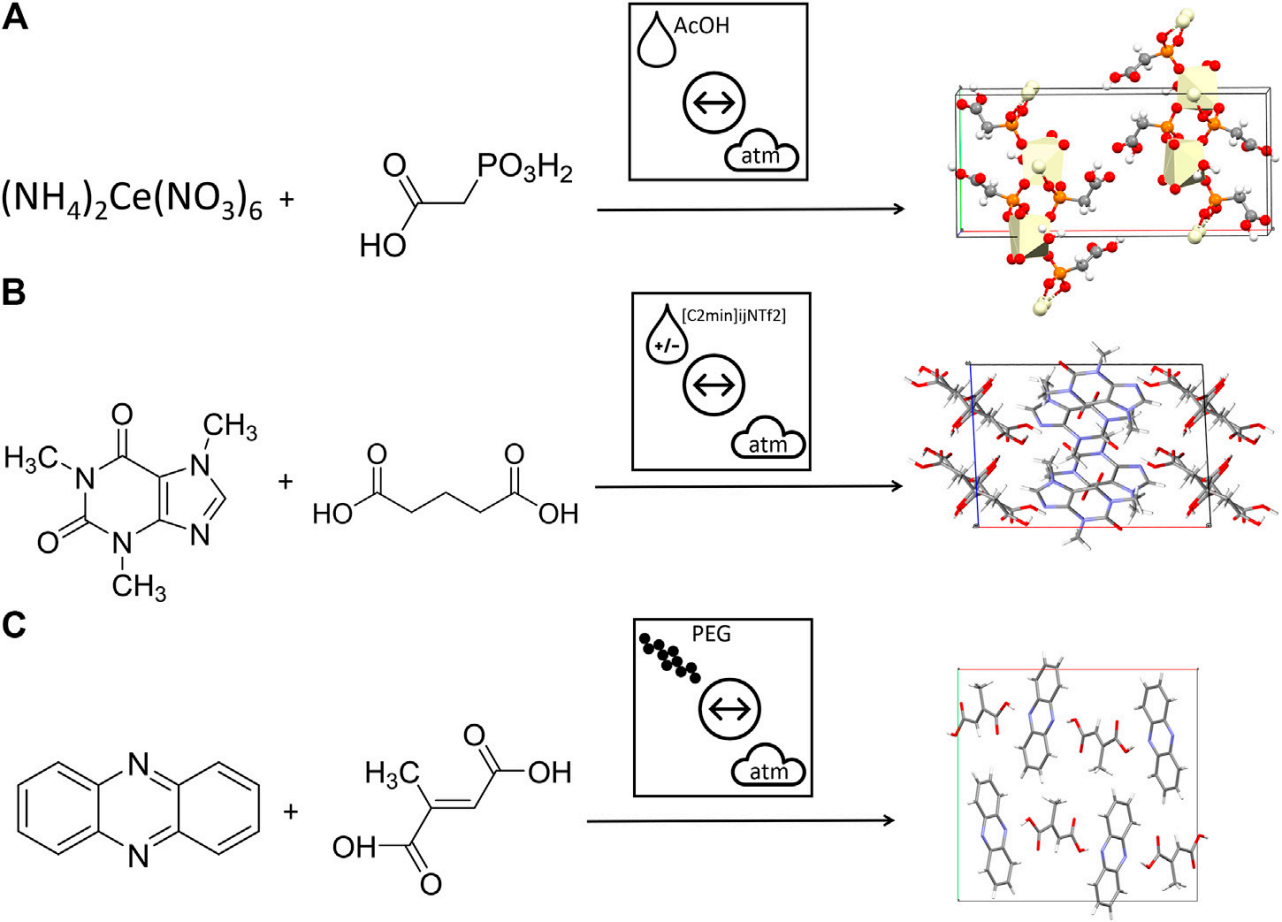
Using the proposed nomenclature to describe mechanochemical transformations with different types of additives. **(A)** An example of liquid assisted grinding formation of Ce based framework (Wilke et al., 2018) **(B)** An example of ionic liquid assisted grinding for the formation of caffeine + glutaric acid cocrystals (Mukherjee et al., 2018) **(C)** An example of polymer assisted grinding for the formation of phenazine + mesaconic acid cocrystals (Hasa et al., 2015).

An additional feature that is missing from current mechanochemical literature is a schematic approach to denote the type of additive being used. This is of primary concern when comparing the effect of the common approaches of LAG, ILAG and POLAG. To demonstrate the strength of the proposed nomenclature, we selected representative examples of each method and show the immediate recognition of these three concepts, schematically, Figure 10. Without the need to deeply consider the text, or indeed understand the physical nature of the additive, the reader is immediately aware how these three vibratory ball milling reactions differ.

**FIGURE 11:**
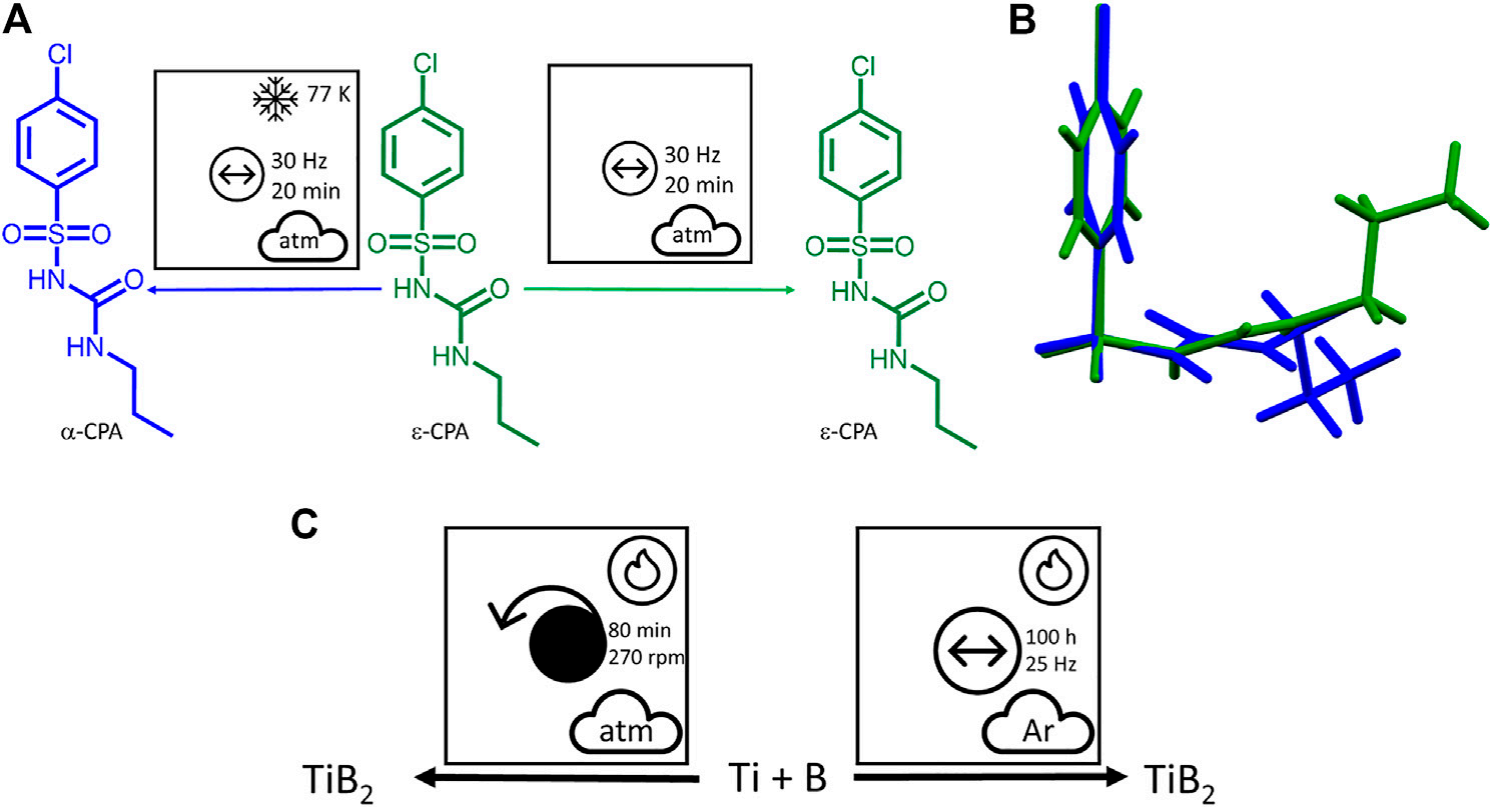
**(A)** Using the proposed nomenclature to describe mechanochemical transformations that differ according to temperature. **(B)** An illustration of the change in molecular conformations on cooling, which is reversible without a mechanical treatment, but is preserved (interlocked) after the molecular layers have been shifted on cryogrinding. **(C)** The self-sustaining mechanosynthesis of TiB_2_ under planetary and vibratory ball milling conditions.

Variation in temperature is also readily visible through use of the proposed nomenclature. This can be exemplified by the effects of cryo-temperatures on milling of ϵ-chlorpropamide, Figure 11 (Drebushchak et al., 2011). As previously discussed, the proposed nomenclature not only provides a thorough understanding of the mechanochemical conditions, but allows the reader immediate recognition of the role of temperature on this polymorphic transformation. The reaction temperature is not always controlled externally, but can also be generated internally from the sample itself as in the case of self-sustaining reactions (Takacs, 2002). In the self-sustaining mechanosynthesis of TiB_2_ for example (Park et al., 1994; Radev and Klisurski, 1994; Takacs, 2002), the amount of time required to ignite the powder depended critically on the type of mechanical action used: 80 min by planetary ball milling, but over 100 h by vibratory ball milling, Figure 11C. This unique origin of reaction temperature is clearly marked using the pictographic approach.

**FIGURE 12:**
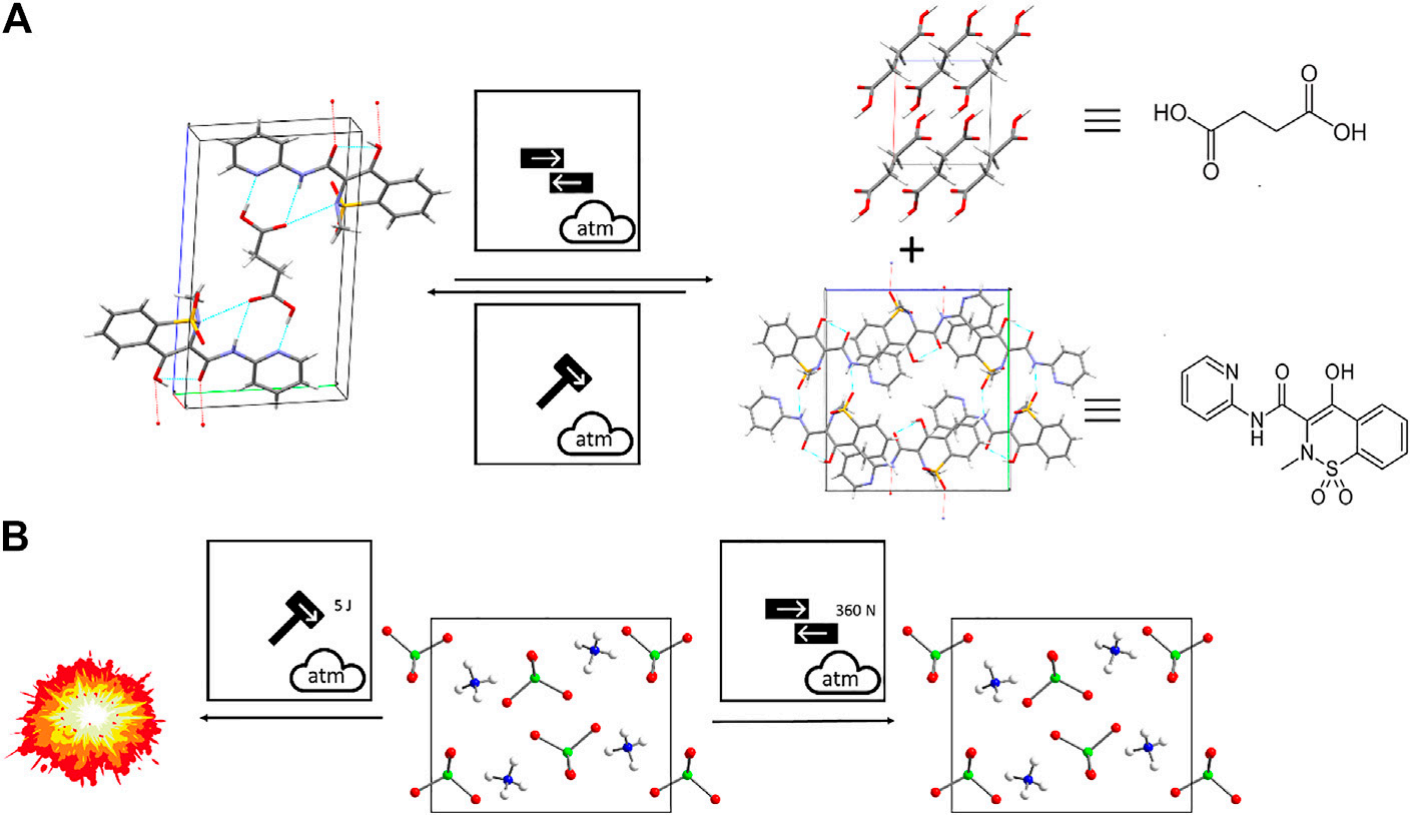
Using the proposed nomenclature to describe mechanochemical transformations that differ according to mechanoreactor. **(A)** Shearing action leads to dissociation of the 2:1 piroxiam:succinic acid cocrystal phase into its coformers, whereas impact action on powder of the coformers leads to formation of the cocrystal. **(B)** Difference in mechanical reactivity of ammonium perchlorate to impact and friction.

The need to specify explicitly the type of mechanical action within mechanochemical protocol is exemplified by the co-crystal formation of piroxicam and succinic acid, Figure 12A (Tumanov et al., 2014). The use of the triple milling ball symbol of Figure 12A would be wholly insufficient in such cases. The differing effects of friction and impact are instead captured explicitly within the proposed system, and a reaction scheme based upon this nomenclature allows immediate recognition of this important mechanochemical phenomenon. The difference in mechanochemical reactivity of energetic materials is also exemplary of the need to make clear the type of mechanical action being applied. Ammonium perchlorate, for example, initiates *ca.* 5 J mechanical impact, but remains insensitive (*i.e.* up to 360 N) when exposed to friction, Figure 12B (Roberts and Royle, 1991).

**FIGURE 13:**
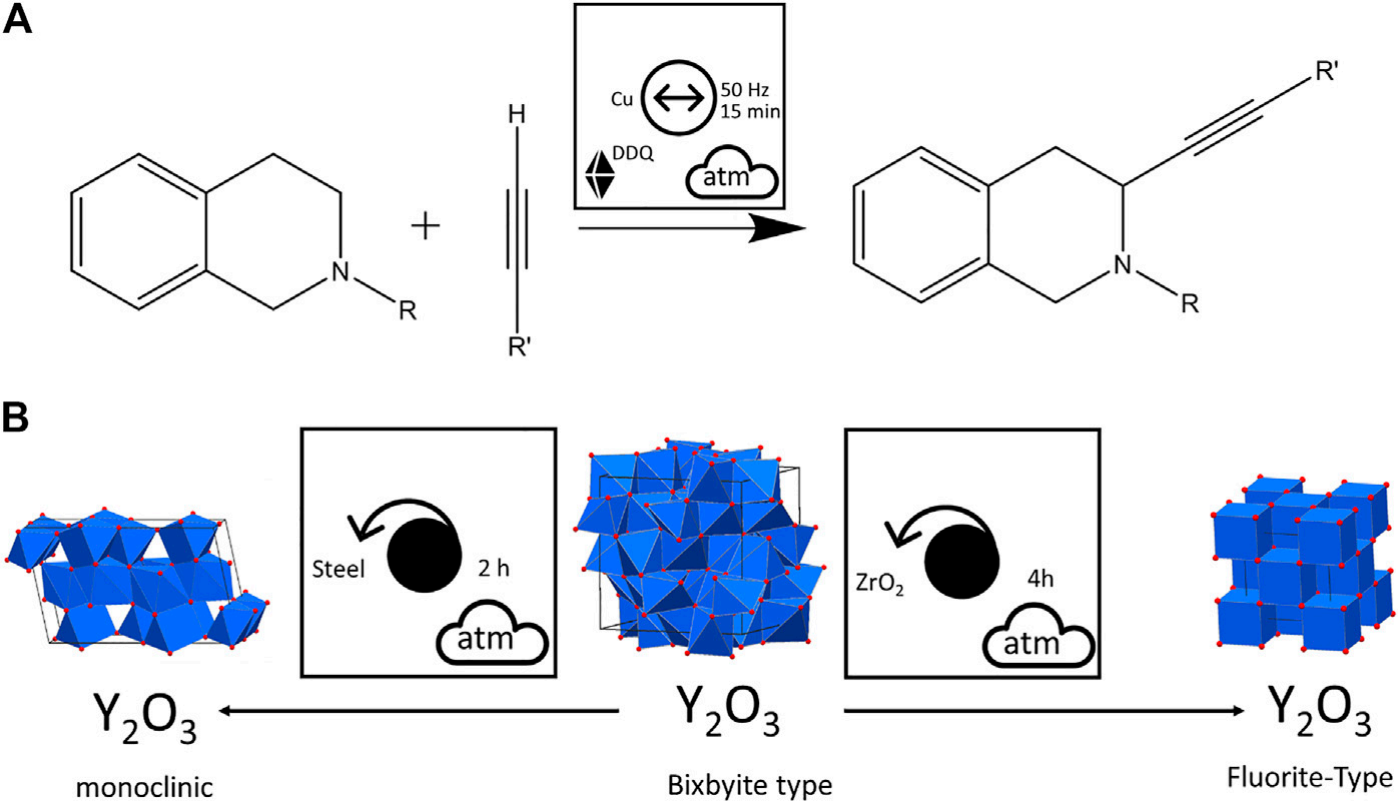
Visualizing the effects of milling ball material on mechanochemical reactions. **(A)** Using the Cu milling balls as catalyst for an oxidative coupling reaction. **(B)** The formation of two unique polymorphic forms of Y_2_O_3_ depending on the material of milling balls during planetary ball milling.

The material of the milling jar and milling bodies can be important for the kinetics, the product composition (Germann et al., 2020) and apparent stability (Kulla et al., 2019), and the very possibility of a transformation on ball-milling. They can act as catalysts of the transformations of the powders which are hit by the balls. Such examples of mechanocatalysis have been documented both for inorganic (Grätz et al., 2020; Li et al., 1999; Chen et al., 2000) and organic (Pickhardt et al., 2020; Haley et al., 2016; Sawama et al., 2018; Cook et al., 2013; Chen et al., 2015; Vogt et al., 2021; Ardila‐Fierro and Hernández, 2021) compounds. For example, the oxidative coupling of tetrahydroisoquinolines with nitromethane by vibratory ball milling with solid additive DDQ was facilitated by using Cu milling balls as catalyst rather than adding another catalytic material to the reaction mixture, Figure 13A (Su et al., 2011). Similarly, the ball milling material can play an important role in the treatment of inorganic phases, as in the case of the polymorphic transformation of Bixbyite (Y_2_O_3_) under planetary ball milling conditions. When milled using steel balls, a monoclinic phase is formed, whereas the Fluorite structure is obtained when ZrO_2_ milling bodies are used, Figure 13B (Begin-Colin et al., 1995). Also the mechanochemical formation of carbon allotropes (fullerenes, carbon nanotubes, carbon onion structures, etc.), which is known to be catalyzed by iron and iron alloys (Zhang et al., 2011; Zhang et al., 2012; Zhang et al., 2014; Velasquez et al., 2016), can be sensitive to the choice of mill (Güler and Evin, 2015), or the substitution of steel milling balls and jar for another material (Surov, 2004). Interestingly, the substitution of steel for ZrO_2_ seemed to have no significant effect on the mechanochemical synthesis of graphene oxide by neat ball milling of pristine graphite (Mahmoud et al., 2018). In all cases involving organic and inorganic reagents, these small details regarding the milling conditions can be again readily observed using the pictographic nomenclature.

**FIGURE 14:**
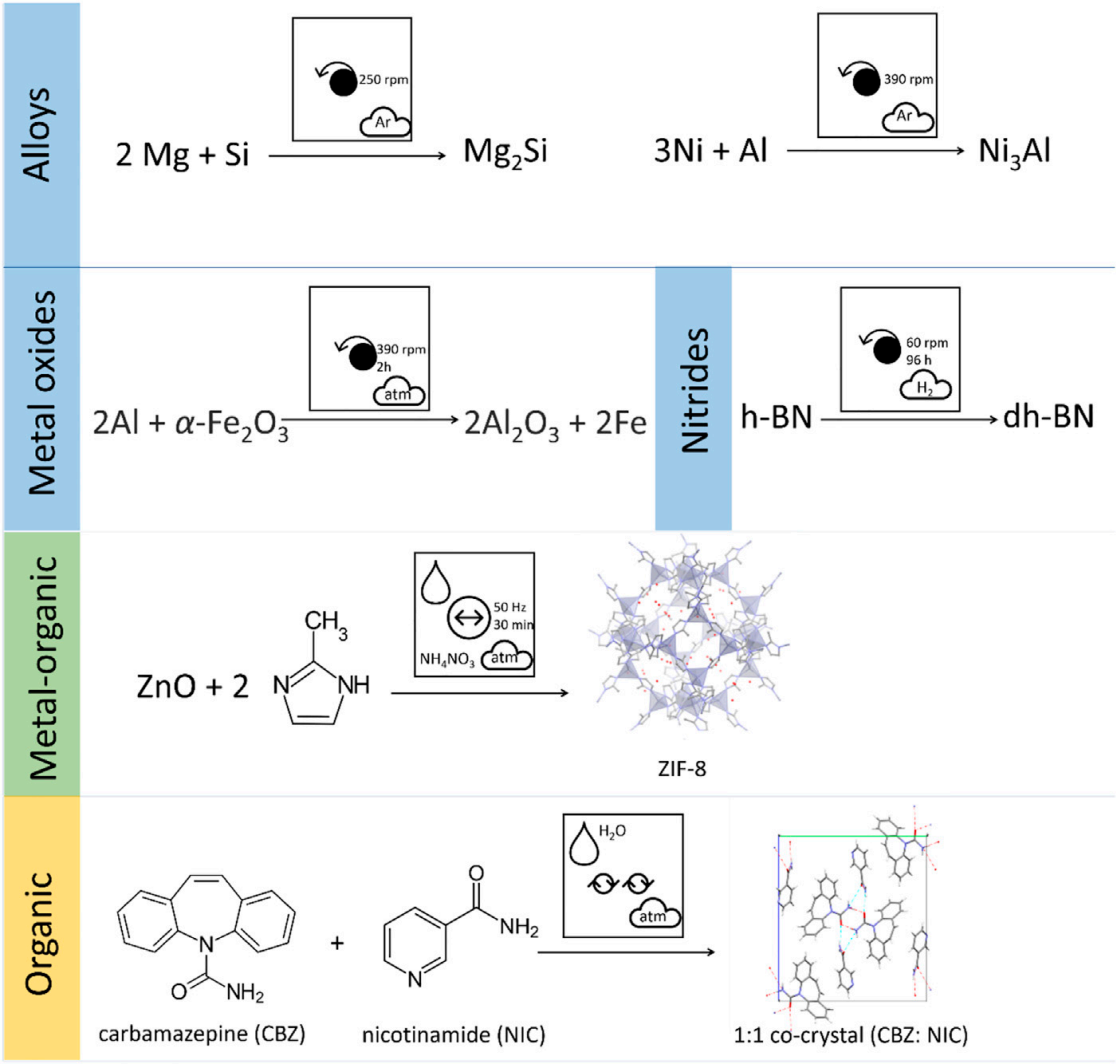
Pictographic representation of literature mechanochemical reactions for a diverse selection of chemical systems, including planetary milling of alloys (Wang and Qin, 2003; Enayati et al., 2004), metal oxides (Stößer et al., 2013), and nitrides (Nash et al., 2016) (e.g. hexagonal boron nitride h-BN converting to defect laden hexagonal boron nitride dh-BN), vibratory ball milling of metal organic frameworks (Batzdorf et al., 2015), and Resonant Acoustic Mixing cocrystal synthesis (Michalchuk et al., 2018a).

Mechanochemical techniques have now been applied to an enormous array of materials syntheses, well beyond what can be covered here. For example, mechanochemical covalent chemical reactions have been reported (Andersen and Mack, 2018b; Bolm and Hernández, 2019; Tan and García, 2019), spanning classical condensation (Haferkamp et al., 2019) or cyclization (Andersen and Mack, 2017) reactions through to metal-catalyzed (Pickhardt et al., 2020) or piezocatalyzed reactions (Kubota et al., 2019). Regarding inorganic materials, syntheses and modifications have been described for hydrated oxides (MacKenzie et al., 1999; Mehrotra et al., 2016; Alex et al., 2020), mixed oxides and ceramics (Balaz et al., 1994; Mackenzie et al., 2000; Temuujin et al., 2000; MacKenzie and Barneveld, 2006), silicates (Temuujin et al., 1998a; Temuujin et al., 1998b; MacKenzie et al., 2007), and high entropy alloys (Kumar et al., 2017; Balcerzak et al., 2019; Kamalakannan et al., 2019), amongst many others. We note that the pictographic representation is equally applicable to any reactions that occur as a result of mechanical action from across all aspects of chemical reactivity, Figure 14. Comparison of the pictographic representations for mechanochemical transformations of very different chemical species reveals immediately the diverse conditions required. It is readily apparent that inorganic compounds tend to be prepared using long duration planetary ball milling conditions under controlled atmospheres, whereas soft materials are prepared by gentler mechanical conditions and often benefit from the addition of liquid additives. Hence, not only does this pictographic representation allows rapid identification of experimental conditions being reported, but also offers a facile approach to identifying trends in experimental conditions successfully applied across the chemical and materials sciences. We therefore expect this clear and concise approach for representing mechanochemical transformations to facilitate new generalisations of mechanochemistry toward targeted and rapid design of new materials and molecular syntheses.

## Summary

The mechanochemical community has expanded significantly in recent years. Throughout most of the 20th century, mechanochemistry was the focus of a smaller and relatively homogeneous scientific community of predominantly solid-state scientists. Now, the community of mechanochemists has flourished, incorporating experts from a wide range of scientific backgrounds. To date, membership in the mechanochemistry field includes researchers from all branches of chemistry, physics, materials sciences, pharmaceutical sciences, biological sciences, and engineering. Many of the newcomers are themselves trained experts in solution or gas phase reactivity, thereby bringing with them many unique viewpoints on phenomena of chemical reactivity. The diversification of the mechanochemical community has triggered new, challenging and fundamentally important scientific questions and has led to mechanochemistry achieving more global impact than ever before.

Mechanochemistry today is understood to include much more than what is strictly defined by IUPAC as a “chemical reaction that is induced by the direct absorption of mechanical energy” (Mechano-Chemical Reaction, 2009). The term mechanochemistry has grown to include any transformation that is observed during or after any type of mechanical treatment, regardless of the exact role of the mechanical action. Moreover, the term mechanochemistry is applied equally to transformations which occur upon stretching of single molecules, through to transformations within and between solids (including those which involve fluid intermediate states). Any transformation that is somehow facilitated by mechanical energy, or reactions that result from thermal- or photochemically induced stress and strain in a solid [the *chemomechanochemical effect* (Boldyrev, 2018)] seem now to be denoted as “mechanochemical”. Thermal or photochemical transformations in solids which have been mechanically pre-treated are similarly denoted as being “mechanically activated”, and hence also fall within the current paradigm of mechanochemistry (Boldyrev, 2018; Galwey, 2020; Shields et al., 2020).

With this growing diversity of the community and the phenomena being explored comes a confusion of scientific languages on the scale of the Tower of Babel. Terminologies and jargon used by experts from one discipline are often misunderstood by experts from a different background. Similarly, much of the over a century’s worth of research in mechanochemistry that is written in the scientific language of 20th century mechanochemical pioneers remains largely incomprehensible to many who enter the field. Moreover, many of these original works have been not digitalized, and are thus not easily accessible until recently. Advances in modern digital technology have made these precious papers and their translations available *via* online platforms, thereby allowing the global community to stand on the shoulders of the ancestral mechanochemical giants. Despite these digital advances, there is still a large miscommunication between the established and emerging mechanochemistry communities. Many well-documented phenomena are unfortunately being regularly re-discovered, with many new terminologies being coined to describe them.

Although in principle, science does not care how it is called, this has the knock-on effect of hindering how the scientific community can discuss, communicate, interpret, search the literature, and hence progress its collective understanding of the field. For example, many who accomplish an organic synthesis in a mechanical device do not realize that they in fact deal not only with a chemical transformation, but with a plethora of tribochemical phenomena. By considering only one aspect of the whole, one risks to miss the elegance that nature has laid before us. For an elephant investigated in parts by blind men may be easily mistaken for a rope, a leaf, or a wall. In this same way, the strict isolation of tribo- and mechanochemistry exists only in the minds of humans.

The growing interest in the mechanochemistry of organic compounds has revealed many new parameters which must be controlled to successfully achieve the reproducible mechanosynthesis of molecules and materials. These new parameters are of course in addition to those that were traditionally considered in tribochemistry and inorganic mechanochemistry. Parameters which are presently known to influence mechanochemical transformations include: the starting polymorph; the size and shape of particles; the type of mechanical action; the atmosphere under which the reaction has occurred; the presence and quantities of additives (solids, liquids, gases, polymers) even if they may not obviously participate in the reaction; the presence of catalysts, including those present as the materials of the milling bodies or reactors. Moreover, the specific parameters associated with the type of mechanical actions (e.g. revolutions per minute in twin screw extrusion or planetary ball milling, or frequency in ball milling, or the number, size and mass of the balls) are certainly important in defining the reaction. Yet, many such parameters are often overlooked, remain unreported, or are difficult to identify in literature reports.

Although efforts at unraveling the mechanistic aspects and driving forces of mechanochemical research are without doubt of central importance, effective communication of protocol and processes do not require such an understanding. It might not be possible to classify unambiguously a reaction as “mechanochemical”, “tribochemical” or “mechanically facilitated thermochemical” without this mechanistic understanding, but we can still impose clarity of communication when reporting our results. Following the successful example of the Crystallographic Information File (CIF), we demonstrate how adopting a standard format for reporting experimental conditions can help ensure that important parameters are both monitored and controlled. Such agreements on the type of information that needs to be presented and how it should be presented so that it is clear to all on first glance, are crucial to unify the community and drive fundamental developments in the field of mechanochemistry.

We believe this *opinion* piece will spark timely and productive discussion across the ever growing and diversifying mechanochemistry community. All symbols displayed in this text are available free of charge for those who wish to use them. Additional pictograms and information will certainly become important as new features of control over mechanochemical reactions emerge, and we as a community must be ready to adapt our nomenclature and standard of practice to accommodate this.

As mechanochemistry becomes an increasingly integral part of efforts to develop environmentally benign strategies for chemical and industrial processes, it is timely to make efforts to unite the dynamic community behind common concepts and definitions in scientific language, and terminology. Only in doing so can we hope to avoid the potential downfall of the “Tower of Babel” and construct a coherent and robust “Tower of Mechanochemistry”. It is only by uniting the community that we can collectively obtain knowledge as, in the words of N. Copernicus, “to know that we know what we know, and that we do not know what we do not know, that is true knowledge.”

## Author's Note

A library of the pictograms used in this study are available free of charge from https://opus4.kobv.de/opus4-bam/frontdoor/index/index/docId/52329

## Abbreviations

ACN, Acetonitrile; AFM, Atomic Force Microscopy; BAM, Bundesanstalt für Materialforschung und -prüfung; BUMS, Bottom Up Mechanochemical Synthesis; CIF, Crystallographic Information File; DAC, Diamond Anvil Cell; DBU, 1,8-Diazabicyclo[5.4.0]undec-7-ene; DDQ, 2,3-Dichloro-5,6-dicyano-1,4-benzoquinone; FAIR, Findable, Accessible, Interoperable, Reusable; FM-PES, Force-Modified Potential Energy Surface; GPa, Giga Pascal; IA-LAG, Inadvertent Liquid Assisted Grinding; IUPAC, International Union of Pure and Applied Chemistry; ILAG, Ionic liquid assisted grinding; IMA, International Mechanochemical Association; LAG, Liquid Assisted Grinding; LVP, Large Volume Press; MOF, Metal-Organic Framework; NG, Neat Grinding; OAD, Oxalic acid dihydrate; PES, Potential Energy Surface; POLAG, Polymer Assisted Grinding; PVP, Polyvinylpyrrolidone; RAM, Resonant Acoustic Mixing or Resonant Acoustic Mixer; RPM, Revolutions per minute; TSE, Twin Screw Extrusion.
